# Psychological, Sociodemographic, and Lifestyle Correlates of Anxiety in Romanian Medical and Nursing Students: A Cross-Sectional Study

**DOI:** 10.3390/healthcare14203455

**Published:** 2026-10-15

**Authors:** Cătălin Pleșea-Condratovici, Constantin-Marinel Vlase, Ionuț-Silviu Vasile, Alina Pleșea-Condratovici, Manuela Arbune, Victor Mihai Pleșea-Condratovici, Răzvan Cosmin Pană

**Affiliations:** 1 Faculty of Medicine and Pharmacy, Research Center in the Medical-Pharmaceutical Field, “Dunarea de Jos” University of Galati, 47 Domneasca Street, 800008 Galati, Romania; catalin.plesea@ugal.ro (C.P.-C.); constantin.vlase@ugal.ro (C.-M.V.); 2 “Dr. Aristide Serfioti” Military Emergency Hospital, 800008 Galati, Romania; manuela.arbune@ugal.ro; 3 Discipline of Endocrinology, Department 5, University of Medicine and Pharmacy of Craiova, 200349 Craiova, Romania; 4 Faculty of Medicine and Pharmacy, “Dunărea de Jos” University of Galaţi, 800008 Galati, Romania; victormihaipc@gmail.com; 5 2nd Department, Faculty of Nursing and Midwifery, University of Medicine and Pharmacy of Craiova, 200349 Craiova, Romania; razvancosminpana@yahoo.com

**Keywords:** anxiety, medical students, nursing students, self-regulation, wellbeing, procrastination, principal component analysis, multiple imputation, mediation analysis, biopsychosocial model

## Abstract

**Highlights:**

**What are the main findings?**

• In 483 Romanian medical and nursing students, 43% screened positive for elevated anxiety (HADS-A ≥ 8), whereas sociodemographic and lifestyle variables (faculty, study year, sex, partner status, living arrangement, siblings, pets, hobbies) showed only small, non-significant associations with anxiety.
• A principal component combining wellbeing, self-esteem, and conscientiousness with low procrastination and neuroticism showed the strongest association with anxiety in every model (β = −0.77; OR = 0.14 per SD); in an exploratory analysis, the procrastination–anxiety association was largely accounted for by wellbeing.

**What are the implications of the main findings?**

• Within the variables measured, psychological characteristics were more strongly associated with anxiety than sociodemographic or lifestyle factors, suggesting that demographic risk profiles alone are unlikely to identify students at risk.
• Longitudinal and intervention studies are needed to establish whether self-regulation and wellbeing represent modifiable targets for reducing anxiety in health-sciences students.

**Abstract:**

**Introduction:** Anxiety is highly prevalent among health-sciences students, yet the relative strength of its sociodemographic, social-contextual, and psychological correlates is rarely examined within a single sample. This cross-sectional study examined the associations of these domains with anxiety in Romanian medical and nursing students within an integrated biopsychosocial framework. **Materials and Methods:** A cross-sectional online survey was conducted with 483 first- to third-year Medicine and Nursing students at the “Dunărea de Jos” University of Galați. Anxiety was measured with the HADS-A. Predictors included sociodemographics, lifestyle variables, the Big Five, Rosenberg Self-Esteem, WHO-5 Wellbeing, and a procrastination scale. Missing data were addressed via multiple imputation. Analyses included pooled correlations, group comparisons, principal component analysis with Promax rotation, hierarchical regression, bootstrapped mediation, and logistic regression. **Results:** Among students with observed scores, 40.8% screened positive for elevated anxiety (HADS-A ≥ 8; 43.0% after imputation). Sociodemographic and lifestyle variables showed small associations that were not statistically significant after adjustment. A principal component combining wellbeing, self-esteem, and conscientiousness with low procrastination and neuroticism showed the strongest association with anxiety across all models. In an exploratory analysis, the procrastination–anxiety association was largely accounted for by wellbeing. **Discussion:** Within the variables measured, psychological characteristics were more strongly associated with anxiety than sociodemographic or lifestyle factors. **Conclusions:** Self-regulation and wellbeing merit attention as potential targets for future longitudinal and intervention research in health-sciences students.

## 1. Introduction

Anxiety is among the most prevalent mental health conditions affecting university students worldwide. Students enrolled in health sciences programmes—medicine and nursing in particular—appear especially vulnerable. Systematic reviews and meta-analyses have documented that approximately one in three medical students reports clinically significant anxiety, a figure that appears to exceed estimates for the general young adult population [1,2]. Nursing students show a comparable burden, with studies across multiple countries converging on elevated anxiety prevalence from the first year of training onwards [3,4]. The consistency of these findings across instruments, settings, and healthcare systems suggests that the excess risk is not merely an artefact of measurement, but reflects something structurally specific to this educational context.

Medical and nursing training places students under a configuration of demands that is qualitatively distinct from other undergraduate experiences. High-volume curricula, high-stakes assessment, and early exposure to clinical environments require students to manage academic pressure alongside the emotional weight of patient contact, witnessed suffering, and the progressive assumption of professional responsibility [2,4]. These stressors are further compounded by the professional culture of both fields, which has historically discouraged the open acknowledgement of psychological distress and may inhibit help-seeking behaviour [5]. The result is a population that is simultaneously highly exposed to anxiety-generating conditions and, in many institutional contexts, poorly supported in managing them.

Research into the determinants of anxiety in this population has grown substantially, yet important gaps remain. Most published studies adopt single-predictor designs that account for modest variance in anxiety outcomes and offer limited guidance for intervention. Comparative investigations that examine medicine and nursing students within the same institution and timeframe—allowing faculty-level differences in curriculum structure, clinical exposure timing, and sociodemographic composition to be disentangled—remain scarce [1,2]. The roles of procrastination and of lifestyle factors such as hobby engagement and pet ownership have received limited attention in health sciences student samples specifically, despite evidence for their relevance in the broader student mental health literature [6,7]. Furthermore, the relationship between academic burnout and anxiety—whether burnout functions as an independent predictor, a consequence, or a mediating pathway between stressors and anxious symptomatology—has not been systematically examined in this context [8,9]. Burnout was included in the questionnaire as a prespecified secondary variable; because its section was added during data collection and was not presented to third-year students, the burnout-related aim could not be pursued (Section 2.4.5).

The present study is organized within the biopsychosocial model [10], which frames health outcomes as the product of biological predispositions, psychological characteristics, and social-contextual conditions acting in concert. Applied to student anxiety, this framework directs attention simultaneously toward dispositional factors—personality traits, self-esteem, and procrastination—alongside social variables such as living arrangement, romantic partnership, and family structure, and behavioural regulators including hobby engagement. Within the psychological domain, neuroticism—the personality trait most consistently associated with anxiety and mood disorders across populations [11]—is of particular theoretical interest. Academic burnout was included as a potential intervening variable between accumulated academic stress and anxiety. This multi-domain perspective addresses the recognized limitation of single-predictor designs and aligns with calls in the medical education literature for theoretically integrated, multivariate approaches to student wellbeing research [1,2].

Among the psychological variables, neuroticism is the trait most consistently associated with anxiety, whereas conscientiousness, through its link with organised and goal-directed behaviour, tends to relate inversely to anxiety; extraversion, agreeableness, and openness show weaker and less consistent associations [11]. Low self-esteem is an established concurrent and prospective correlate of anxiety symptoms [12], low subjective wellbeing overlaps substantially with negative affect, and procrastination has been conceptualised as a failure of emotion regulation that increases negative affect [7,13]. Among the contextual variables, living alone, the absence of a partner, and the absence of leisure activities such as hobbies were expected to be associated with higher anxiety, in line with the stress-buffering role attributed to social support and leisure engagement [14,15], whereas pet ownership has been proposed as a source of companionship and emotional support [16]. These expectations guided the selection of variables, but the study did not test directional hypotheses for individual predictors. Against this background, the present study examined anxiety—as measured by the Hospital Anxiety and Depression Scale, Anxiety subscale (HADS-A)—in a sample of first- to third-year Medicine and Nursing students enrolled at the same university. We hypothesized that neuroticism and procrastination would be positively associated with anxiety, and self-esteem and subjective wellbeing inversely associated with it. The study was not preregistered; an initially planned analysis of academic burnout as an intervening variable could not be carried out (Section 2.4.5), and the mediation model reported in Section 3.7 was specified after inspection of the data and is presented as exploratory. By integrating sociodemographic, social, and psychological correlates within a hierarchical regression framework—and applying principal component analysis to address multicollinearity among correlated psychological variables—the study aimed to describe the anxiety burden in this population and to compare the strength of association of sociodemographic, social, and psychological correlates.

## 2. Materials and Methods

### 2.1. Study Design and Ethical Approval

This was a cross-sectional observational study conducted during the 2024–2025 academic year at the University “Dunărea de Jos” of Galați, Faculty of Medicine and Pharmacy, Romania. The study adhered to the principles of the Declaration of Helsinki and applicable national regulations. Ethical approval was granted by the “Dunărea de Jos” University of Galați in collaboration with the Ethics Committee of the Galați College of Physicians (No. 1165/29.11.2024). All participants provided electronic informed consent prior to completing the questionnaire, and participation was entirely voluntary. Responses were confidential rather than anonymous: participants entered their name and date of birth, which served to prevent duplicate submissions; these identifiers were removed before analysis and are not included in the public dataset.

### 2.2. Participants and Sampling

The target population comprised students enrolled in Years 1, 2, and 3 of the Medicine and Nursing programmes at the above institution. No exclusion criteria were applied beyond enrolment in the specified years and programmes: all enrolled students are adults, follow instruction in Romanian, and were able to give informed consent; ages below 18 were treated as invalid entries (see below), and duplicate submissions were prevented as described in Section 2.1. Participants were recruited through convenience sampling: the questionnaire link was distributed via WhatsApp group messages to all students enrolled in Years 1–3 of Medicine and Years 1 and 3 of Nursing, and the questionnaire was completed voluntarily; second-year Nursing students were not reached. No incentives were offered. Exact enrolment figures for each cohort were not available to the research team, so a response rate could not be calculated. No responses were excluded (participant flow: Supplementary Table S13). The initial dataset comprised *n* = 483 participants (Medicine: *n* = 364, 75.4%; Nursing: *n* = 119, 24.6%). Prior to analysis, all scale scores were checked against the theoretical range of the respective instrument, and values outside that range were recoded to missing: two procrastination scores (>90), two RSES scores (>30), and one HADS-A score (>21). Five age values below 18 were likewise treated as implausible for university enrolment and recoded to missing. All recoded values were handled via multiple imputation together with the originally missing data (see Section 2.6.2); no value was retained in any analysis after being classified as invalid. A priori power analysis was conducted using G*Power (version 3.1.9.7, Heinrich Heine University Düsseldorf, Düsseldorf, Germany) for linear multiple regression (fixed model, R^2^ deviation from zero) with 12 predictors, assuming α = 0.05, statistical power = 0.80, and a small-to-medium effect size (f^2^ = 0.10), yielding a minimum required sample of *n* = 201. For principal component analysis (PCA) stability, a minimum of *n* = 250 Is recommended [17]. The final sample comfortably exceeded both thresholds.

### 2.3. Data Collection

Data were collected via an online questionnaire administered through PsyToolkit (version 3.6.0; https://www.psytoolkit.org, accessed on 6 October 2026) [18], an open-access platform for psychological survey research. The questionnaire was designed in Romanian and included all sociodemographic items and psychometric instruments described below. Completion took approximately 20–25 min. Data were collected in December 2024, during the teaching period. The questionnaire link was distributed sequentially to the cohorts rather than to all students at once, and the questionnaire was revised during data collection: the HADS-A was added after the first cohort (third-year Nursing) had responded, and the hobby item and the burnout section were added after the third-year cohorts had responded. The revised questionnaire was re-sent to the cohorts that had already responded, but few students completed it a second time. As a result, hobby engagement and burnout are unobserved for all third-year students and HADS-A for all third-year Nursing students; this missingness is by design and depends only on programme and study year (Supplementary Table S1). Within each version, items were presented in a fixed order, and no item was mandatory, so participants could leave any question unanswered. The questionnaire could be completed on a computer or a mobile device, and no attention-check items were included.

### 2.4. Measures

#### 2.4.1. Anxiety—Hospital Anxiety and Depression Scale, Anxiety Subscale (HADS-A)

The HADS-A is a widely validated 7-item self-report instrument assessing anxiety symptoms over the preceding week, with each item scored on a 0–3 scale (total range 0–21) [19]. Established cut-offs classify scores as normal (0–7), borderline (8–10), or scores in the ≥11 screening range, indicating probable anxiety according to the HADS-A threshold (≥11) [20]. The HADS-A served as the primary dependent variable in all analyses. The Romanian wording administered was a translation prepared by the research team, as described in Section 2.4.7.

#### 2.4.2. Personality—Big Five Inventory (BFI)

Personality was assessed using a Romanian-language adaptation of the short form of the Big Five Inventory (BFI-S) [21,22], comprising 15 items (3 per dimension) rated on a 1–7 Likert scale (1 = strongly disagree to 7 = strongly agree). The five dimensions assessed were Neuroticism, Extraversion, Openness to Experience, Agreeableness, and Conscientiousness. Each dimension score is calculated as the mean of its three items, with higher scores indicating a stronger presence of the trait.

#### 2.4.3. Self-Esteem—Rosenberg Self-Esteem Scale (RSES)

Global self-esteem was assessed using the Rosenberg Self-Esteem Scale (RSES) [23], a 10-item instrument scored on a 0–3 Likert scale (total range 0–30). Higher scores indicate greater self-esteem. The RSES is among the most widely used and psychometrically robust measures of self-esteem in research settings.

#### 2.4.4. Wellbeing—WHO-5 Wellbeing Index

Subjective wellbeing was measured with the WHO-5 Wellbeing Index [24], a 5-item scale assessing positive mood, vitality, and general interest over the previous two weeks. Raw scores (0–25) are multiplied by four to yield a percentage score (0–100), with scores below 50 indicating poor wellbeing and scores below 28 suggesting likely depression.

#### 2.4.5. Academic Burnout—School Burnout Inventory (SBI-U9)

Academic burnout was assessed using the School Burnout Inventory—University version (SBI-U9) [25], a 9-item instrument yielding three subscale scores—Exhaustion, Cynicism, and Inadequacy—as well as an Overall Burnout composite score. Items are rated on a 6-point Likert scale. The SBI-U9 has been validated for use in university student populations and demonstrates sound factorial validity and internal consistency.

The burnout section was added to the questionnaire after the third-year cohorts had responded (Section 2.3), so it was never presented to the 161 third-year students; of the 322 first- and second-year students who received it, 243 (75.5%) completed it (50.3% of the total sample). Because burnout was thus unobserved for an entire study year and the burnout-related aim required comparisons across years, it was not imputed and was excluded from the multivariate analyses, and the original hypothesis involving burnout could not be tested (Section 4.7). Students who completed the burnout section did not differ from the remaining students in observed HADS-A scores (M = 6.66, SD = 4.26, *n* = 243 vs. M = 7.17, SD = 4.19, *n* = 132; Welch t = −1.11, *p* = 0.266, d = −0.12).

#### 2.4.6. Procrastination—General Procrastination Scale, Student Version (GPS-S)

Procrastination was assessed with the General Procrastination Scale (GPS) [26], a 20-item trait measure rated on a 5-point scale (1 = extremely uncharacteristic of me, 5 = extremely characteristic of me), of which 10 items are reverse-keyed. Owing to an error in building the online questionnaire in PsyToolkit, two items of the original scale (item 15, “I often have a task finished sooner than necessary”, reverse-keyed, and item 17, “I usually buy even an essential item at the last minute”) were omitted; the omission was noticed only after data collection had ended. The 18 administered items (original items 1–14, 16 and 18–20), of which nine are reverse-keyed, were scored according to the original key and summed to a total score (theoretical range 18–90), with higher scores indicating greater procrastination. The items were translated into Romanian by the research team (Section 2.4.7); the full Romanian version administered, with the original item numbers and the reverse-keyed items marked, is provided in Supplementary Material S16. Because of the omission, scores are not directly comparable with those of the full 20-item scale, and the version has no validation evidence beyond the internal consistency reported in Section 2.4.7.

#### 2.4.7. Internal Consistency in the Present Sample

All instruments were administered in Romanian. As no validated Romanian versions were available to the research team, the items of all instruments were translated from English by two members of the research team (A.P.-C. and C.P.-C.), both proficient in English (CEFR level C1, C2 in reading), who compared their translations and agreed on a final wording; no back-translation, cognitive debriefing or pilot testing was performed, and the translations have not been formally validated. Measurement equivalence with the original instruments can therefore not be assumed (Section 4.7), and internal consistency in the present sample is reported below as the only available psychometric evidence for these versions.

Internal consistency was estimated in the present sample for every multi-item score used in the analyses, from item-level responses of participants with complete data on the respective scale, after exclusion of the out-of-range cases described in Section 2.2. Cronbach’s α and McDonald’s ω are reported in Supplementary Table S12. α was computed from the raw item covariances, and ω is omega total from a one-factor model estimated by iterated principal-axis factoring on the item covariance matrix, with items treated as continuous. Ninety-five percent confidence intervals were obtained by non-parametric percentile bootstrap (1000 resamples of participants). No item-level imputation was performed. Reliability was good for the HADS-A (α = 0.84, 95% CI 0.81–0.86; ω = 0.85, 0.82–0.87), the RSES (α = 0.87, 0.84–0.89; ω = 0.87, 0.85–0.89), the WHO-5 (α = 0.86, 0.83–0.89; ω = 0.86, 0.84–0.89), and the GPS-S (α = 0.83, 0.80–0.85; ω = 0.84, 0.81–0.86). As expected for three-item scales covering broad traits, the BFI-S dimensions showed lower values (neuroticism α = 0.63, ω = 0.67; extraversion α = 0.59, ω = 0.65; openness α = 0.76, ω = 0.76; agreeableness α = 0.56, ω = 0.60; conscientiousness α = 0.55, ω = 0.56); the implications are addressed in Section 4.7.

### 2.5. Sociodemographic and Lifestyle Variables

The questionnaire included items assessing: faculty (Medicine vs. Nursing), study year (1, 2, or 3), age, sex, living arrangement, sibling status and birth order, pet ownership and attachment, romantic partner status, and hobby engagement. Living arrangement was a multiple-response item, recoded into four mutually exclusive categories: (1) parental or extended family home, (2) romantic cohabitation, (3) peer cohabitation (shared flat), and (4) living alone. The 22 participants who selected more than one option were assigned to the first applicable category in this order (20 selected family home and romantic cohabitation and were coded as family home; one selected family home and living alone and was coded as family home; one selected romantic and peer cohabitation and was coded as romantic cohabitation). The binary ‘lives alone’ indicator used in the regression models therefore identifies participants who selected living alone and no other arrangement. Sibling status was recoded into a binary variable (only child vs. has siblings), with a secondary ordinal variable capturing birth order (youngest, middle/mixed, oldest, twin/same age) for those with siblings. Pet ownership was operationalized both as an ordinal relationship scale (1 = never had and dislikes pets to 6 = currently owns and deeply attached) and as a binary current ownership variable.

### 2.6. Statistical Analysis

#### 2.6.1. Descriptive Statistics and Normality

The study and the analysis plan were not preregistered. The descriptive, correlational, group-comparison, principal component, regression, and logistic analyses were planned before data analysis; the mediation model was specified after inspection of the correlation structure and is reported as exploratory. Means, standard deviations, medians, and frequency distributions were computed for all study variables. Distributions were examined graphically (histograms and Q–Q plots) and through skewness and kurtosis values; normality of raw variables was not treated as a prerequisite, and model-specific diagnostics are reported for the regression models (Supplementary Table S9 and Figure S1). HADS-A scores were further categorised per established cut-offs and prevalence rates reported by subgroup.

#### 2.6.2. Missing Data and Multiple Imputation

Missing data were handled using Multiple Imputation by Chained Equations (MICE) [27], implemented in the miceforest Python package (v6.0.5) (LightGBM models (v4.5.0; Microsoft Corporation, Redmond, WA, USA) with predictive mean matching, 5 donor candidates). The imputation model included all 19 variables used in the analyses: age, sex, faculty, study year, living arrangement, sibling status and birth order, pet relationship, partner status, hobby engagement, procrastination, the five BFI-S dimensions, self-esteem, wellbeing, and HADS-A; the outcome was imputed together with the predictors. Sex, faculty, and sibling status were treated as categorical; ordinal variables were imputed as numeric and rounded to the nearest valid category. M = 20 imputed datasets were generated with 10 iterations per chain (random seed 42). Convergence was evaluated through mean-convergence plots, and observed and imputed distributions were compared visually for all imputed variables. Missingness on HADS-A and hobby engagement resulted from the questionnaire revisions described in Section 2.3 and was therefore missing by design, in that whether these variables were observed depended on programme and study year, both included in the imputation model. This makes the missing-at-random assumption plausible for these variables, although it remains an untestable assumption: participants without an observed HADS-A score also differed from those with one in age and procrastination (Supplementary Table S2), variables that were likewise included in the imputation model. Because no third-year Nursing student had an observed HADS-A score, no observed prevalence can be reported for this subgroup, and any model-based estimate for it depends entirely on the imputation specification; such estimates are therefore not reported as prevalence. Burnout was excluded from the imputation model (Section 2.4.5). All pooled estimates were derived using Rubin’s rules [28], and the fraction of missing information (FMI) is reported for each model parameter.

#### 2.6.3. Correlational Analysis

Pooled Pearson correlations were computed across M = 20 imputed datasets and combined using Rubin’s rules, yielding a 10 × 10 correlation matrix covering all continuous predictors and HADS-A. Statistical significance was evaluated with Bonferroni correction applied across all pairwise comparisons (α corrected = 0.05/45 = 0.0011). Results are visualised as a heatmap.

#### 2.6.4. Group Comparisons

Differences in HADS-A scores across categorical variables were examined using Welch’s independent-samples *t*-test (faculty, sex, pet ownership, hobby engagement, sibling status) and one-way ANOVA (study year, partner status, living arrangement). All tests were computed in each of the M = 20 imputed datasets and pooled using Rubin’s rules. Effect sizes are reported as Cohen’s d for two-group comparisons and η^2^ for multi-group comparisons. For two-group comparisons, the pooled mean difference with its 95% confidence interval is also reported; effect sizes were computed in each imputed dataset and averaged. Bonferroni correction was applied across all comparisons; none reached corrected significance (all uncorrected *p* > 0.05).

#### 2.6.5. Principal Component Analysis

PCA was conducted on eight continuous psychological predictors: Procrastination, Openness, Conscientiousness, Extraversion, Agreeableness, Neuroticism, Self-Esteem (RSES), and Wellbeing (WHO-5). Age was excluded as a demographic rather than a psychological variable. Burnout was excluded due to excessive missing data.

Sampling adequacy was confirmed via the Kaiser–Meyer–Olkin statistic (KMO = 0.78, exceeding the recommended threshold of 0.60) and Bartlett’s Test of Sphericity (χ^2^(28) = 876.1, *p* < 0.001). The number of components to retain was determined by parallel analysis (95th percentile criterion; [29]), which identified two components—a more conservative and empirically defensible criterion than the Kaiser eigenvalue-greater-than-one rule [30].

The final solution was estimated once, on the across-imputation mean of the eight variables, standardised to z-scores (i.e., on their correlation matrix), and its stability was then checked by repeating the PCA in each of the 20 imputed datasets: Tucker congruence coefficients between each imputation-specific solution and the final solution were ≥0.998 for both components. Component scores were then computed in each of the 20 imputed datasets by applying the fixed regression-method weights of this solution, so that the component definition was identical across imputations; scores were standardised (M = 0, SD = 1).

Three rotation solutions were compared—unrotated, Varimax (orthogonal), and Promax (oblique)—to evaluate structural clarity and theoretical coherence. Promax rotation was selected as the final solution, given moderate but non-negligible inter-component correlations (PC1–PC2: r = 0.40), which precluded the assumption of component independence required by Varimax. The two components were labelled based on their loading patterns:

• PC1—Self-Regulation & Wellbeing: high loadings from Wellbeing (WHO-5), Self-Esteem and Conscientiousness; strong negative loadings from Neuroticism and Procrastination.
• PC2—Openness & Sociability: high loadings from Openness, Extraversion, Agreeableness and Conscientiousness.

Promax-rotated component scores were used as predictors in the subsequent regression analyses, which reduces the collinearity among the eight correlated psychological measures; because Promax components are themselves correlated, variance inflation factors were nevertheless checked in all regression models.

#### 2.6.6. Hierarchical Multiple Regression

HADS-A scores were predicted via hierarchical multiple regression in three sequential blocks, with predictors entered as follows:

• Block 1—Sociodemographic: Age, Sex (female), Faculty (Nursing), Study Year, Lives Alone.
• Block 2—Social and Lifestyle: Has Partner, Has Siblings, Has Pet, Has Hobby.
• Block 3—Psychological (PCA components): PC1 (Self-Regulation & Wellbeing), PC2 (Openness & Sociability).

Binary predictors were coded 0/1 with the following reference categories: sex (male), faculty (Medicine), living arrangement (lives with others), partner status (no current partner; the three partnered categories were combined), sibling status (only child), pet ownership (no current pet), and hobby engagement (no hobby). Study year was entered as a linear term (1–3); a sensitivity model with year as two dummy variables (Year 2 and Year 3 vs. Year 1) is reported in Supplementary Table S8. Age was entered in years.

For each block, we report R^2^, ΔR^2^, and the F-change test, together with unstandardised coefficients (B, in HADS-A points), standardised coefficients (β), pooled standard errors, Barnard–Rubin degrees of freedom, 95% confidence intervals, *p*-values, and the fraction of missing information (FMI). Coefficients and standard errors were pooled across the 20 imputed datasets using Rubin’s rules [28]; R^2^ was averaged across imputations, and F-change tests were pooled with the D2 procedure, as for the group comparisons (Section 2.6.4). Model assumptions were assessed in each imputed dataset through residual-versus-fitted and Q–Q plots, the Breusch–Pagan test, Ramsey’s RESET test, and Cook’s distances (Supplementary Table S9, Figure S1); because the Breusch–Pagan test indicated heteroscedasticity in part of the imputed datasets, the final model was also re-estimated with heteroscedasticity-consistent (HC3) standard errors for all coefficients (Supplementary Table S15). Variance inflation factors (VIF) were computed to assess multicollinearity. As a sensitivity analysis, the regression and logistic models were re-estimated with the eight psychological scales entered as separate standardised predictors instead of the component scores (Supplementary Table S14).

#### 2.6.7. Mediation Analysis

As an exploratory analysis, a simple mediation model (Hayes Model 4 logic [31]) was estimated with procrastination as the predictor, subjective wellbeing (WHO-5) as the intervening variable, and HADS-A as the outcome, all standardised. The model was specified after inspection of the correlation structure and is therefore exploratory rather than confirmatory. In each imputed dataset, the paths a, b, c, c′ and the indirect effect a × b were estimated by OLS, and their standard errors were obtained by non-parametric bootstrap (2000 resamples); estimates and bootstrap standard errors were then combined across the 20 imputed datasets with Rubin’s rules, following the multiple-imputation bootstrap procedure. Ninety-five percent confidence intervals are reported for every path. The model was also re-estimated with age, sex, faculty, and study year as covariates (Supplementary Table S11). Given the cross-sectional design, the model decomposes concurrent associations and does not establish temporal or causal ordering.

#### 2.6.8. Anxiety Prevalence and Logistic Regression

Using HADS-A cut-offs, the prevalence of borderline (8–10) and clinical (≥11) anxiety was reported for the sample, and the prevalence of elevated anxiety (≥8) was reported by faculty and study year.

A pooled binary logistic regression was conducted with screen-positive anxiety (HADS-A ≥ 8) as the outcome. This threshold combines the borderline (8–10) and the ≥11 screening ranges (pooled prevalence 43.0%), which provides a sufficient number of events for stable logistic regression estimation. The same 11-predictor structure as the final linear regression model was used. Age, study year, and the component scores were standardised; binary predictors were coded as in Section 2.6.6. Coefficients were pooled on the log-odds scale across the 20 imputed datasets using Rubin’s rules and exponentiated to odds ratios (ORs) with 95% CIs. Model performance was summarised by Nagelkerke’s pseudo-R^2^ (averaged across imputations, as a measure of explained variation), discrimination by the area under the ROC curve (AUC; pooled with its standard error across imputations), and calibration by the calibration slope and intercept and the Hosmer–Lemeshow test. Optimism in the AUC and calibration slope was estimated by bootstrap (200 resamples). Events per variable are reported.

#### 2.6.9. Sensitivity Analyses

To assess the influence of outcome imputation, the final regression and logistic models were re-estimated (i) in the imputed datasets restricted to participants with an observed HADS-A score (n = 375) and (ii) in complete cases with no missing values on any model variable (*n* = 277). Prevalence estimates are reported both for observed cases (Wilson 95% CI) and pooled across imputations (Rubin’s rules). Participants with and without an observed HADS-A score were compared on all other variables (Supplementary Table S2).

#### 2.6.10. Software and Significance Threshold

All analyses were conducted in Python (v3.10.11) using the following packages: pandas (v3.0.2), numpy (v2.4.4), scipy (v1.17.1), scikit-learn (v1.8.0), miceforest (v6.0.5), pingouin (v0.6.1), statsmodels(v0.15.0), and matplotlib (v3.10.9)/seaborn (v0.13.2). Pooled regression and logistic regression estimates followed Rubin’s rules as implemented via custom routines consistent with established multiple imputation practice [32]. The significance threshold was α = 0.05 (two-tailed) throughout, with Bonferroni correction applied where multiple simultaneous comparisons were conducted. All figures were produced at ≥300 dpi.

## 3. Results

### 3.1. Sample Characteristics

A total of 483 students enrolled in the first three years of Medicine and Nursing programmes at the Dunărea de Jos University of Galați completed the questionnaire. All descriptive statistics reported in this section are computed on the observed (non-imputed) data; the denominator for each variable is the number of cases with that variable observed, and the extent of missingness is reported explicitly.

The sample comprised 75.4% Medicine students (*n* = 364) and 24.6% Nursing students (n = 119). Of the 482 students reporting sex, 77.2% identified as female (*n* = 372) and 22.8% as male (n = 110); one participant did not declare sex. The mean age was 25.1 years (SD = 9.6, range 18–54, *n* = 477), reflecting both the typical undergraduate population and a smaller cohort of mature students predominantly enrolled in Nursing. The year-of-study distribution comprised 43.3% first-year (n = 209), 23.4% second-year (*n* = 113), and 33.3% third-year (*n* = 161) students; the Nursing programme did not include second-year participants in this sample. Sociodemographic and lifestyle characteristics are summarised in Table 1.

Observed means, standard deviations, and skewness/kurtosis values for the psychological scales are presented in Table 2. Subjective wellbeing (WHO-5; M = 65.1, SD = 19.8, *n* = 412) and self-esteem (RSES; M = 20.8, SD = 5.7, *n* = 429) showed typical levels for student samples. The Big Five factors had means in the moderate-to-high range (range of means: 4.19 for neuroticism to 5.41 for agreeableness, all *n* = 443). The mean procrastination score was 46.9 (SD = 10, *n* = 466).

The mean HADS-A score among students with observed values (n = 375) was 6.84 (SD = 4.24, range 0–19), with a slightly right-skewed distribution (skewness = 0.37, kurtosis = −0.48) consistent with previous reports in health-sciences students. Applying the standard HADS-A thresholds to the observed cases, 59.2% scored within the normal range (0–7; n = 222), 19.2% in the borderline range (8–10; n = 72), and 21.6% in the ≥11 screening range (n = 81), yielding a combined elevated-anxiety prevalence of 40.8% in the observed sample (Figure 1). HADS-A was missing for 108 cases (22.4%); these values were addressed via multiple imputation for the inferential analyses, as described in Section 3.2.

### 3.2. Missing Data and Multiple Imputation

Missingness by variable and by programme–year group is shown in Supplementary Table S1. It was below 3% for sociodemographic items and between 8% and 15% for the psychological scales, but was structural for two variables, as a consequence of the questionnaire revisions described in Section 2.3: hobby engagement was not observed for any third-year student (35.6% missing overall), and HADS-A was not observed for any third-year Nursing student (22.4% missing overall; 57.1% within Nursing). Compared with participants with an observed HADS-A score, those without were more often Nursing students, older, in a higher study year, and reported higher procrastination, but did not differ on sex, social variables, personality, self-esteem, or wellbeing (Supplementary Table S2). Burnout (49.7% missing) was excluded as described in the Methods. Out-of-range values (Section 2.2) were recoded to missing before imputation. Twenty imputed datasets were generated via MICE with predictive mean matching, run for 10 iterations per chain. Diagnostic inspection confirmed satisfactory convergence (all chains stabilised within two to three iterations) and good preservation of observed marginal distributions. The pooled mean of HADS-A across imputations (M = 6.97) was within 0.15 points of the observed mean (M = 6.84), and the fraction of missing information ranged from 0.005 to 0.049 across continuous variables, indicating that m = 20 was sufficient for stable inference. 

### 3.3. Correlational Structure

Pearson correlations among all continuous predictors and the HADS-A outcome were computed in each of the 20 imputed datasets and pooled via Fisher’s z-transformation with Rubin’s rules using the Barnard–Rubin small-sample degrees-of-freedom correction. After Bonferroni correction for 45 comparisons (α = 0.0011), HADS-A scores correlated most strongly with subjective wellbeing (r = −0.63, *p* < 0.001), neuroticism (r = +0.56, *p* < 0.001), and self-esteem (r = −0.55, *p* < 0.001), all in the medium-to-large effect range. Procrastination (r = +0.35, *p* < 0.001) and conscientiousness (r = −0.31, *p* < 0.001) showed moderate associations, and age a small one (r = −0.18, *p* < 0.001). Extraversion (r = −0.15, *p* = 0.001), agreeableness (r = −0.15, *p* = 0.001), and openness (r = −0.11, *p* = 0.016) did not survive Bonferroni correction. The full pooled correlation matrix is presented in Figure 2.

Several inter-predictor correlations were substantial and informative for subsequent multivariate analyses. Procrastination correlated negatively with conscientiousness (r = −0.54), and wellbeing correlated positively with self-esteem (r = +0.58), suggesting clusters of correlated measures.

### 3.4. Group Differences in Anxiety

Group comparisons of HADS-A scores were conducted across the eight categorical variables (faculty, study year, sex, hobby engagement, pet ownership, sibling status, partner status, and living arrangement), all planned a priori, with each test computed in all 20 imputed datasets and pooled using Rubin’s rules (Welch’s *t*-tests for two-group comparisons; one-way ANOVA pooled via the D2 method for multi-group comparisons). After Bonferroni correction (α = 0.0063), no comparison reached statistical significance. Anxiety did not differ between Medicine and Nursing students (t(141.2) = 0.92, *p* = 0.360, d = 0.10), across study years (F(2, 464.3) = 1.04, *p* = 0.355, η^2^ = 0.008), or by sex (t(196.4) = −1.78, *p* = 0.077, d = −0.18). Comparable non-significant findings emerged for hobby engagement, pet ownership, sibling status, partner status, and living arrangement (Table 3).

Three non-significant trends are nonetheless worth noting because they bear on the discussion. Anxiety scores increased numerically across study years (M = 6.59, 7.05, and 7.42 for Years 1, 2, and 3, respectively), were descriptively highest among students living alone (M = 8.35) and lowest among those in romantic cohabitation (M = 5.78), and were modestly elevated among students reporting no hobby engagement (d = −0.20, *p* = 0.068). None of these patterns survived statistical testing. The complete set of group means and comparisons is depicted in Figure 3. Group differences on demographic and social-contextual variables were thus small, in contrast with the moderate-to-large correlations observed for the psychological variables in Section 3.3.

### 3.5. Component Structure of the Psychological Predictors: Principal Component Analysis

Principal component analysis was conducted on the eight continuous predictors estimated on the across-imputation mean of the standardised variables. Repeating the PCA in each of the 20 imputed datasets yielded near-identical loadings (Tucker’s congruence with the final solution ≥ 0.998 for each component) and small across-imputation variability in loadings (mean loading SD = 0.013), indicating that the component structure was stable across imputations.

Sampling adequacy was good (KMO = 0.78), and Bartlett’s test of sphericity was highly significant (χ^2^(28) = 867.1, *p* < 0.001), confirming the suitability of the data for component extraction. Parallel analysis (95th-percentile criterion, 1000 random datasets) and the Kaiser criterion converged on a two-component solution (Figure 4). The two components accounted for 52.9% of the total variance (eigenvalues 3.04 and 1.19). Promax (oblique) rotation was selected over Varimax because the components were intercorrelated (r = 0.40, exceeding the 0.32 threshold that justifies oblique rotation) and yielded a clearer simple structure. Because the components are correlated, the variance attributable to each after rotation is not uniquely partitioned; sums of squared structure loadings are reported in Supplementary Table S6.

The first component (PC1), labelled Self-Regulation and Wellbeing, was defined by positive pattern coefficients for wellbeing (λ = 0.76), self-esteem (λ = 0.70), and conscientiousness (λ = 0.46), and negative coefficients for neuroticism (λ = −0.76) and procrastination (λ = −0.65). The second component (PC2), Openness and Sociability, was defined by openness (λ = 0.78), extraversion (λ = 0.73), agreeableness (λ = 0.53), and conscientiousness (λ = 0.51). Neuroticism showed a secondary pattern coefficient of 0.36 on PC2, and conscientiousness loaded on both components (.46 on PC1, 0.51 on PC2); communalities ranged from 0.39 (agreeableness) to 0.65 (conscientiousness). The full pattern and structure matrices are given in Table 4 and Supplementary Table S6. The component labels are descriptive summaries of the loading pattern and do not imply validated latent constructs.

Principal component extraction on the correlation matrix, Promax rotation; entries are pattern (regression) coefficients. Component correlation: PC1–PC2 r = 0.40. Because components are correlated, variance is reported from the unrotated solution; structure coefficients, communalities, and post-rotation sums of squares are in Supplementary Table S6. Projecting participants into the component space and colouring by HADS-A category revealed a clear visual gradient along PC1: students in the normal-anxiety range clustered toward the high pole, whereas students in the borderline and ≥11 screening ranges concentrated at the low pole (Figure 5). The distribution of PC1 scores shifted systematically downward across the three anxiety categories (Normal: median ≈ +0.46; Borderline: median ≈ −0.35; Clinical: median ≈ −0.93), foreshadowing the regression findings reported in Section 3.6.

### 3.6. Predictors of Anxiety: Hierarchical Multiple Regression

A three-block hierarchical multiple regression predicting HADS-A scores was fitted in each of the 20 imputed datasets, with standardised coefficients and variance estimates pooled using Rubin’s rules. Block 1 entered sociodemographic variables (age, sex, faculty, study year, lives-alone indicator), Block 2 added social and lifestyle variables (partner status, sibling status, pet ownership, hobby engagement), and Block 3 added the two Promax-rotated PCA component scores.

Block 1 explained a small but significant proportion of variance (R^2^ = 0.062, pooled F-change(5, 1338) = 5.54, *p* < 0.001). Block 2 did not add significant incremental variance (ΔR^2^ = 0.016, F-change(4, 1515) = 1.79, *p* = 0.13). Block 3 produced a substantial increment, accounting for an additional 44.7% of variance (ΔR^2^ = 0.447, F-change(2, 432) = 177.7, *p* < 0.001) and raising the total explained variance to R^2^ = 0.526. Coefficients for all three blocks are given in Supplementary Table S7.

In the final model, the dominant predictor was PC1 (Self-Regulation and Wellbeing): each one-SD increase was associated with a 3.12-point lower HADS-A score (B = −3.12, 95% CI [−3.43, −2.80]; β = −0.77, 95% CI [−0.84, −0.69], *p* < 0.001), a large inverse association. PC2 (Openness and Sociability) showed a small positive association (B = 0.47, 95% CI [0.16, 0.79]; β = 0.12, *p* = 0.003) that was retained with heteroscedasticity-consistent standard errors (*p* = 0.004). Once the psychological components were entered, none of the sociodemographic or lifestyle predictors reached significance (all *p* ≥ 0.054), and the psychological block accounted for the majority of the explained variance (Table 5b, Figure 6). Within Block 3, the unique contribution of PC1 (increase in R^2^ when added last) was 0.419, compared with 0.010 for PC2. Entering study year as a categorical rather than linear term did not alter the conclusions for the psychological components; the Year 3 versus Year 1 contrast reached nominal significance (B = −0.66, *p* = 0.049), consistent with the marginal linear term (Supplementary Table S8). Residual diagnostics indicated approximately normal residuals with no influential observations (maximum Cook’s D = 0.03) and no evidence of misspecification (RESET median *p* = 0.45), but the Breusch–Pagan test indicated heteroscedasticity in part of the imputed datasets (median *p* = 0.040, range 0.014–0.207; Supplementary Table S9, Figure S1). The final model was therefore re-estimated with HC3 robust standard errors for all coefficients (Supplementary Table S15): the estimates, confidence intervals, and conclusions were unchanged (PC1: B = −3.12, SE = 0.16, *p* < 0.001; PC2: B = 0.47, SE = 0.16, *p* = 0.004; no sociodemographic or lifestyle predictor significant). All variance inflation factors were below 1.7.

**Table 5:** (**a**). Hierarchical regression block summary: R^2^, ΔR^2^, and F-change tests. (**b**). Final hierarchical regression model predicting HADS-A anxiety (Block 3; m = 20 pooled): unstandardised (B, HADS-A points) and standardised (β) coefficients.

(**a**)						
**Block**	**R^2^**	**ΔR^2^**	**F-Change**	**df1**	**df2**	***p***
Block 1: Sociodemographic	0.062	0.062	5.54	5	1338	<0.001
Block 2: + Social/Lifestyle	0.079	0.016	1.79	4	1515	0.128
Block 3: + PCA Components	0.526	0.447	177.65	2	423	<0.001
(**b**)						
**Predictor**	**B**	**SE**	**95% CI**	**β**	***p***	**FMI**
Age (years)	0.01	0.02	[−0.03, 0.05]	0.03	0.531	0.19
Sex (female vs. male)	0.51	0.36	[−0.21, 1.22]	0.05	0.163	0.08
Faculty (Nursing vs. Medicine)	0.36	0.41	[−0.44, 1.17]	0.04	0.377	0.18
Study year (per year)	−0.32	0.17	[−0.65, 0.01]	−0.07	0.054	0.12
Lives alone (vs. with others)	0.77	0.51	[−0.23, 1.77]	0.05	0.130	0.08
Has partner (vs. none)	0.51	0.30	[−0.08, 1.10]	0.06	0.091	0.07
Has siblings (vs. only child)	−0.04	0.31	[−0.65, 0.58]	0.00	0.908	0.10
Owns pet (vs. none)	−0.50	0.28	[−1.04, 0.04]	−0.06	0.070	0.05
Has hobby (vs. none)	0.01	0.32	[−0.62, 0.64]	0.00	0.978	0.10
PC1: Self-Regulation & Wellbeing (per SD)	−3.12 ***	0.16	[−3.43, −2.80]	−0.77	<0.001	0.10
PC2: Openness & Sociability (per SD)	0.47 **	0.16	[0.16, 0.79]	0.12	0.003	0.06

R^2^ averaged across the 20 imputed datasets; F-change tests and degrees of freedom pooled with the D2 procedure, hence df2 may exceed N. Total R^2^ = 0.526; all VIFs ≤ 1.65. SE, CI, and *p* from Rubin’s rules with Barnard–Rubin df; FMI = fraction of missing information. ** *p* < 0.01, *** *p* < 0.001.

The final model was robust to the handling of missing outcomes: restricting the analysis to participants with an observed HADS-A score (n = 375) or to complete cases (n = 277) yielded virtually identical coefficients for PC1 (β = −0.76 and −0.75, respectively) and for PC2 (β = 0.14 and 0.12) and the same pattern of non-significant sociodemographic and lifestyle effects (Supplementary Table S4).

As a further sensitivity analysis, the eight psychological scales were entered as separate standardised predictors instead of the two components (Supplementary Table S14). Neuroticism (β = 0.40, 95% CI [0.33, 0.47]), wellbeing (β = −0.39, [−0.47, −0.31]) and self-esteem (β = −0.24, [−0.32, −0.16]) were independently associated with HADS-A, whereas procrastination and conscientiousness were not once the other scales were adjusted for; all VIFs were ≤2.2 and R^2^ = 0.595. The corresponding logistic model gave the same pattern (Supplementary Table S14). The PCA-based models were retained as the primary analyses because the eight scales are substantially intercorrelated (Figure 2) and the study aim was to compare domains rather than individual scales.

### 3.7. Exploratory Mediation Analysis: Procrastination, Wellbeing, and Anxiety

As an exploratory decomposition of the cross-sectional associations among procrastination, wellbeing, and anxiety, a simple mediation model was tested (Procrastination → Wellbeing → HADS-A). Standardised path coefficients and bootstrap standard errors were estimated in each imputed dataset and pooled with Rubin’s rules.

Procrastination was negatively associated with wellbeing (a = −0.38, 95% CI [−0.46, −0.29]), and wellbeing was negatively associated with anxiety controlling for procrastination (b = −0.58, 95% CI [−0.65, −0.50]). The total association between procrastination and anxiety (c = +0.35, 95% CI [0.26, 0.44]) was reduced when wellbeing was added to the model (c′ = +0.13, 95% CI [0.05, 0.22]). The indirect component was a × b = 0.22 (95% CI [0.16, 0.27]), corresponding to 62% of the total association (Table 6, Figure 7). Adjusting for age, sex, faculty, and study year gave similar values (a × b = 0.19, 95% CI [0.13, 0.24]; 56%; Supplementary Table S11). Hence, a substantial share of the procrastination–anxiety association was statistically accounted for by wellbeing; given the cross-sectional design, this pattern is descriptive and does not establish temporal or causal ordering.

### 3.8. Screen-Positive Anxiety: Prevalence and Logistic Regression

Among participants with an observed HADS-A score, 40.8% (95% CI 35.9–45.8) screened positive for elevated anxiety (≥8); the pooled estimate across imputed datasets was 43.0% (95% CI 38.4–47.5). Observed prevalence was similar across programme–year groups (Table 7, Figure 8); for third-year Nursing students, no observed scores were available and the subgroup estimate was not reported. A binary logistic regression predicting elevated anxiety from the full predictor set was fitted in each imputed dataset; coefficients were pooled on the log-odds scale via Rubin’s rules and exponentiated to odds ratios (ORs). The number of events ranged from 199 to 212 across imputed datasets (mean 208; 18.9 events per variable). Nagelkerke’s pseudo-R^2^ averaged 0.46. Discrimination was good (pooled AUC = 0.85, 95% CI 0.81–0.89; optimism-corrected AUC = 0.83) and calibration adequate (optimism-corrected calibration slope = 0.91; Hosmer–Lemeshow median *p* = 0.43 across imputations; Supplementary Table S10).

In the final model (Table 8, Figure 9), PC1 (Self-Regulation and Wellbeing) was again the dominant predictor: each one-standard-deviation increase was associated with an 86% lower odds of elevated anxiety (OR = 0.14, 95% CI [0.09, 0.22], *p* < 0.001). PC1 is a standardised, sample-derived score, so this is a conditional association rather than a risk reduction. Pet ownership was associated with lower odds of elevated anxiety (OR = 0.55, 95% CI [0.34, 0.92], *p* = 0.022); however, because this effect was not detected in any of the continuous analyses and would not survive Bonferroni correction, it should be regarded as exploratory and treated with caution. PC2 (Openness and Sociability) was associated with somewhat higher odds (OR = 1.42, 95% CI [1.04, 1.92], *p* = 0.026), an association that, like that of pet ownership, would not survive correction for multiple testing. No other demographic or lifestyle predictor was significantly associated with the binary outcome (all *p* ≥ 0.065).

Odds ratios were similar when the model was restricted to participants with observed HADS-A scores or to complete cases (PC1: OR = 0.16 and 0.17, respectively; Supplementary Table S5).

## 4. Discussion

### 4.1. Overview of Findings

This cross-sectional study of 483 first- to third-year Medicine and Nursing students at the “Dunărea de Jos” University of Galați examined the demographic, social, and psychological correlates of anxiety (HADS-A) within an integrated biopsychosocial framework. Three findings define the substantive contribution. First, the prevalence of elevated anxiety in the observed sample was high (40.8% scoring at or above the borderline threshold of HADS-A ≥ 8), with 21.6% of students scoring in the ≥11 screening range. Second, none of the sociodemographic or lifestyle variables—faculty, study year, sex, partner status, living arrangement, sibling status, pet ownership, or hobby engagement—distinguished anxious from non-anxious students at conventional significance levels after Bonferroni correction. Third, a principal component we labelled Self-Regulation and Wellbeing showed the strongest association with anxiety in every multivariate model, accounting for most of the explained variance in continuous HADS-A scores and for substantially lower odds of screen-positive anxiety. Within the variables measured, psychological characteristics were therefore more strongly associated with anxiety than sociodemographic or social-contextual factors.

The 40.8% elevated-anxiety prevalence observed here exceeds the global meta-analytic estimate of 33.8% in medical students reported by Quek and colleagues [1], as well as the lifetime and 12-month rates documented in European general-population epidemiological surveys [33,34], although these figures refer to diagnosed disorders over longer time frames and are not directly comparable with a screening proportion based on symptoms in the preceding week. These findings are also consistent with the recent trend of elevated psychiatric symptoms (more precisely depressive symptoms) observed in our recent work (34.0% with PHQ-9 ≥ 10) in this student population [35].

### 4.2. Demographic and Social Correlates

Group differences across the eight categorical variables were small (d = 0.04–0.20) and not statistically significant. Although the sample was adequately powered for the omnibus regression model, the confidence intervals around these effect sizes do not exclude small effects, and the contextual domain was represented by a limited set of self-reported indicators; the findings should therefore be read as an absence of evidence for moderate or large effects rather than as evidence of no effect.

The non-significant sex difference contrasts with the well-documented female preponderance in anxiety disorders in general populations [33,36], but is consistent with the WHO World Mental Health International College Student Project’s documentation that prevalence patterns in highly selected student samples can differ from those in the general population [37]. Whether this reflects characteristics of the student population, the screening instrument, or sampling cannot be determined from the present data. Differences by faculty and by year of study were likewise small and not statistically significant. Although our descriptive means rose numerically from Year 1 to Year 3, this trend did not reach significance and was eliminated entirely in the multivariate model. The present design cannot distinguish curriculum-related from individual sources of this variation.

The null findings for social-contextual variables—partner status, living arrangement, pet ownership, sibling status, and hobby engagement—are perhaps the most theoretically surprising. Social support theory predicts that closer relational ties and structured leisure activities should buffer against anxiety [14]. Three of the four descriptive trends in our data are directionally consistent with this expectation (lower anxiety among those in romantic cohabitation, with hobby engagement, and with pet ownership), but none was statistically significant, either in the univariable comparisons or after adjustment for the psychological components. One possibility, which the present design cannot test, is that social context is associated with anxiety through its relationship with psychological functioning; alternatively, the coarse indicators available here may simply have been insufficient to capture contextual influences.

### 4.3. The Self-Regulation and Wellbeing Component as the Dominant Predictor

The most consistent finding of this study is that a principal component combining high wellbeing, self-esteem, and conscientiousness with low procrastination and neuroticism (PC1) showed the strongest association with anxiety across every analytic strategy tested. In the hierarchical regression, the unique variance attributable to PC1 (the increase in R^2^ when PC1 is added to a model containing all other predictors, 0.419) was several times larger than the variance explained by the demographic and lifestyle blocks together (R^2^ = 0.079), and its standardized coefficient was in the large-effect range. In the logistic regression, each standard-deviation increase in PC1 was associated with 86% lower odds of elevated anxiety.

The clustering of self-regulatory and wellbeing measures on a single component is consistent with integrative theoretical accounts in which trait neuroticism, conscientiousness, and subjective wellbeing share substantial common variance. The strong negative loadings of neuroticism and procrastination, alongside positive loadings of wellbeing and self-esteem, are consistent with Sirois and Pychyl’s [7] model in which procrastination represents a failure of emotion regulation that erodes wellbeing and elevates negative affect over time, and with Steel’s [13] meta-analytic conceptualisation of procrastination as quintessential self-regulatory failure.

If confirmed longitudinally, the pattern would be consistent with the rationale for broad-spectrum self-regulation and wellbeing interventions in student health services, rather than approaches targeting a single trait. Mindfulness-based programmes have shown efficacy in increasing resilience to stress in university students, including in randomised controlled trials with rigorous designs [38], and target self-regulatory and emotion-regulation skills that overlap with the measures loading on this component.

The small positive association between the second component, Openness and Sociability, and anxiety (β = 0.12) should be interpreted cautiously. Openness, extraversion, and agreeableness were negatively rather than positively correlated with anxiety at the bivariate level (r = −0.11 to −0.15), and neuroticism had a secondary coefficient of 0.36 on this component; the association therefore emerges only after adjustment for PC1 and probably reflects shared variance with neuroticism rather than a risk associated with sociability itself. We regard it as exploratory.

### 4.4. Procrastination, Wellbeing, and the Mediation Pathway

In the exploratory mediation analysis, a substantial share of the procrastination–anxiety association was statistically accounted for by subjective wellbeing, with a smaller residual direct path. This partial-mediation pattern aligns with temporal motivation theory and self-regulation failure models, in which delaying valued tasks generates short-term negative effects that accumulate into broader wellbeing decrements and downstream anxiety [7,13].

Should this pattern be replicated in longitudinal data, it would be relevant to intervention design: procrastination is frequently targeted with behaviour-focused strategies (implementation intentions, time-management training), and complementing such strategies with components that build emotion-regulation skills might be worth testing. This is consistent with intervention work showing that training emotion-regulation skills can reduce procrastination, supporting an emotion-focused rather than purely behavioural conceptualisation of the problem [39].

### 4.5. The Exploratory Role of Pet Ownership

Pet ownership was associated with elevated anxiety only in the binary logistic regression, where it was linked to 45% lower odds of elevated anxiety. Importantly, this effect was absent in the continuous correlation, group comparison, and linear regression analyses, and it would not survive Bonferroni correction within the logistic model. We therefore treat it as exploratory.

Replication in larger, longitudinal samples is needed before any interpretation is warranted.

### 4.6. Methodological Strengths

Several methodological features increase confidence in the present findings. First, multiple imputation with twenty datasets and the fraction-of-missing-information criterion ensured efficient handling of missingness while propagating its uncertainty into all inferential analyses [27]. Missingness on the outcome was concentrated in identifiable programme–year groups and was therefore accounted for by variables in the imputation model, and sensitivity analyses restricted to observed outcomes and to complete cases yielded the same conclusions. Second, the principal component analysis combined parallel analysis, oblique Promax rotation justified by the inter-component correlation pattern, and a cross-imputation robustness check (Tucker’s congruence ≥ 0.998) indicating a stable component structure. Third, all inferential procedures employed Rubin’s pooling rules with Barnard–Rubin small-sample degrees-of-freedom corrections, and the mediation analysis combined bootstrap standard errors across imputations in the same framework [40].

### 4.7. Limitations

Several limitations should temper the interpretation of these findings. First, the cross-sectional design prevents causal inference; the directional specification of the exploratory mediation model (procrastination → wellbeing → anxiety) reflects theoretical priors rather than demonstrated temporal precedence, and the reverse pathway (anxiety eroding wellbeing and increasing procrastination) is equally plausible. Maxwell and Cole [41] have shown that cross-sectional approaches to mediation can produce substantially biased estimates of longitudinal parameters; longitudinal designs with at least three measurement waves are needed to disambiguate causal directionality.

Second, all measures are self-reported, which introduces shared-method variance that may inflate associations between psychological predictors and the HADS-A outcome [42]. Future research should incorporate clinician-rated diagnostic interviews and biobehavioural anxiety markers (e.g., heart rate variability, cortisol) to triangulate findings.

Third, the sampling strategy was convenience-based and drawn from a single university, limiting generalizability. The faculty imbalance (75% Medicine, 25% Nursing) and the absence of Nursing Year 2 students constrain comparisons between the two programmes. Because enrolment figures for each cohort were not available, a response rate could not be computed; moreover, the questionnaire contained no attention-check items, so careless responding cannot be ruled out. HADS-A missingness was concentrated in the Nursing subsample, where only first-year students (51 of 119) provided observed scores; Nursing estimates therefore rest partly on imputation and depend on the missing-at-random assumption and on the imputation specification; sensitivity analyses restricted to observed outcomes yielded the same conclusions for the main models, but no observed prevalence estimate is available for third-year Nursing students. Replication in stratified multi-site samples, ideally including health-sciences students from multiple Romanian universities and from other Central and Eastern European contexts, would strengthen external validity.

Fourth, burnout was excluded from the primary analyses because the burnout section was added to the questionnaire during data collection and was never presented to third-year students, so that it was unobserved for an entire study year; the burnout-related aim stated in the Introduction could therefore not be pursued. Fifth, the HADS-A is a screening rather than diagnostic instrument, and the 21.6% rate of scores ≥ 11 should not be interpreted as a clinical-diagnosis prevalence. Diagnostic confirmation through structured clinical interviews is needed before policy-relevant epidemiological claims can be advanced.

Sixth, several further limitations apply to the multivariable results. Residual confounding by unmeasured variables cannot be excluded, and the contextual domain was represented by a small set of coarse self-reported indicators, so the comparison between psychological and contextual correlates is asymmetric. The measures loading on PC1, particularly neuroticism and current wellbeing, overlap conceptually with anxiety, which may inflate the association through shared content rather than shared cause. Convenience recruitment and nonresponse may have introduced selection bias, and the component structure and regression models were derived and evaluated in the same sample, so their performance is likely to be optimistic. Finally, the number of analyses conducted increases the risk of chance findings, and the exploratory results should be interpreted accordingly. The Romanian versions of all instruments were translated by the research team without back-translation and have not been formally validated; although internal consistency was good for all scales, measurement equivalence with the original instruments cannot be assumed. The three-item BFI-S scales had modest internal consistency (α = 0.55–0.76), which attenuates their associations and adds measurement error to the components on which they load. The procrastination scale was administered with two of its 20 items inadvertently omitted, which limits comparability with studies using the full scale.

### 4.8. Practical Implications

The present findings are hypothesis-generating. Within the variables measured, psychological characteristics were more strongly associated with anxiety than demographic or social-contextual factors, which suggests that demographic risk profiles alone are unlikely to be sufficient for identifying students at risk. Whether self-regulation and wellbeing constitute modifiable targets for reducing anxiety—as suggested by intervention studies targeting emotion-regulation skills in students [38,39]—and whether assessing them adds value to direct anxiety screening are questions that require longitudinal, multisite, and intervention studies before institutional decisions about screening or resource allocation can be informed by these data.

## 5. Conclusions

In this cross-sectional study of medical and nursing students at a single Romanian university, a substantial proportion of participants screened positive for elevated anxiety. Sociodemographic and lifestyle variables showed small associations with anxiety that were not statistically significant after adjustment, whereas a principal component capturing self-regulation, wellbeing, self-esteem, conscientiousness, and the inverse of procrastination and neuroticism showed a large inverse association across all models. An exploratory analysis indicated that much of the procrastination–anxiety association was statistically accounted for by wellbeing. These findings identify self-regulation and wellbeing as candidate targets for future research and require replication in longitudinal, multisite samples before informing screening or intervention policy.

## Figures and Tables

**Figure 1:**
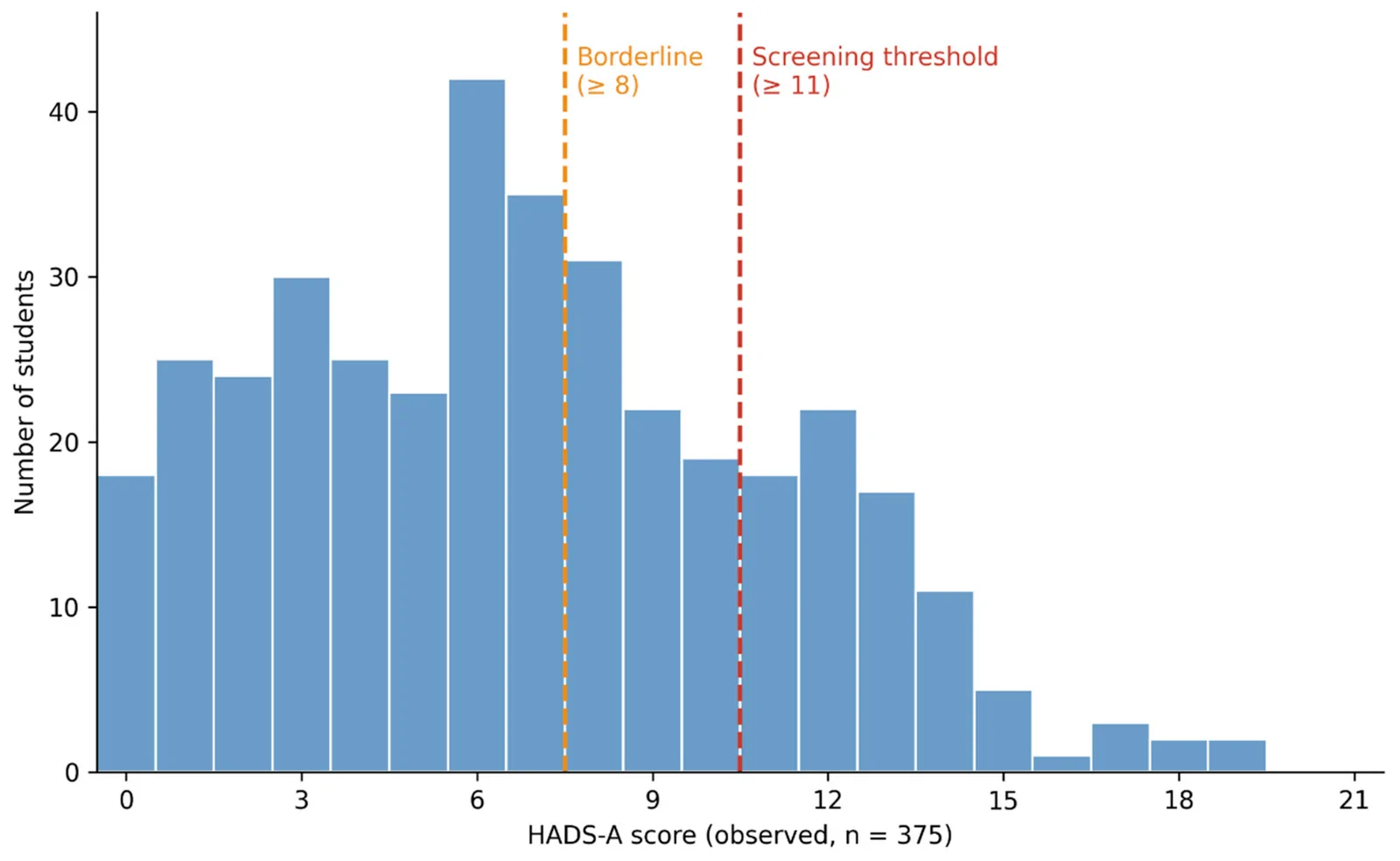
Distribution of HADS-A anxiety scores in the sample, with the borderline (≥8) and ≥11 screening thresholds.

**Figure 2:**
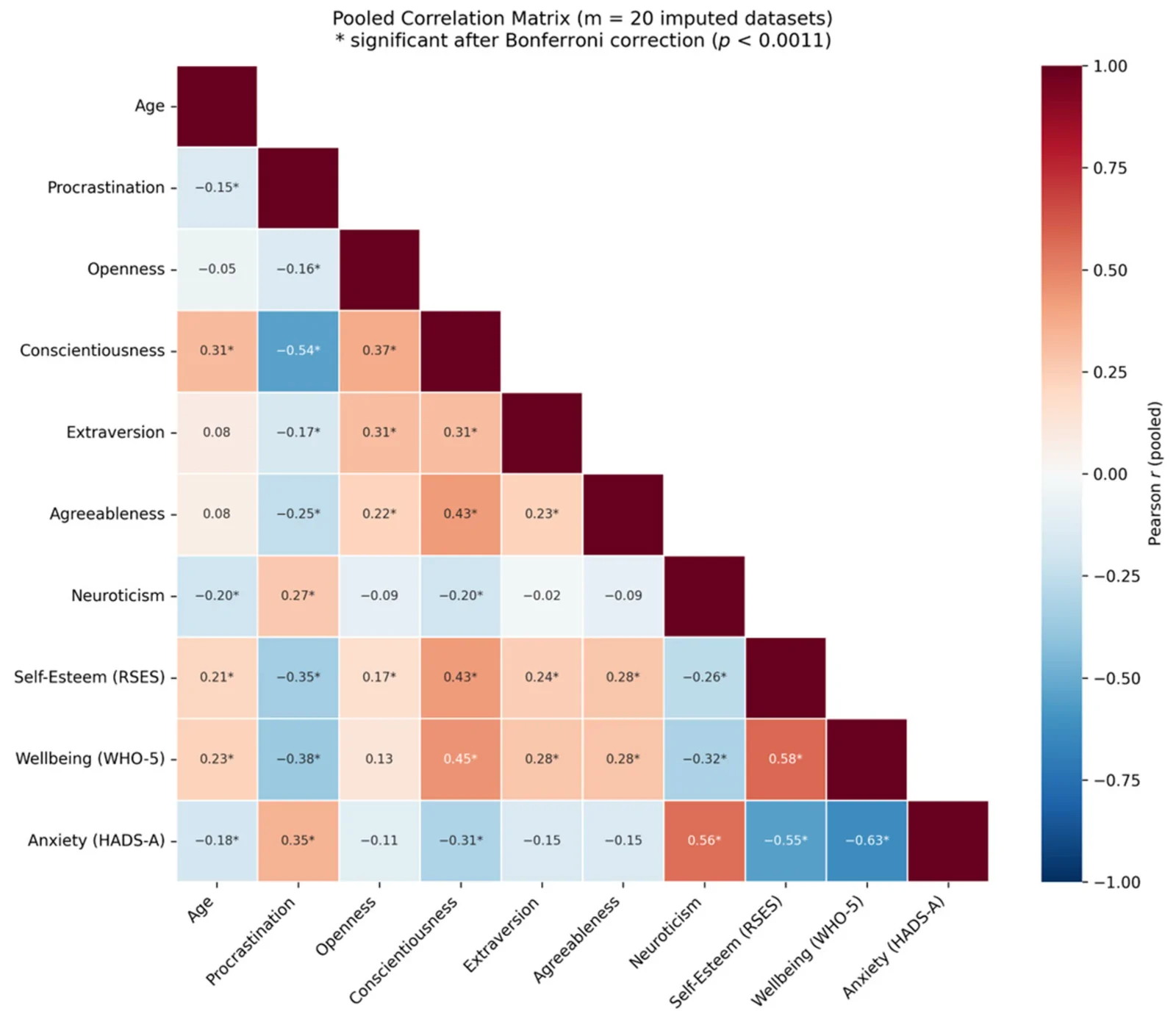
Pooled correlation matrix among continuous predictors and HADS-A across 20 imputed datasets. Coefficients in cells are pooled Pearson r; asterisks mark correlations significant after Bonferroni correction for 45 comparisons (*p* < 0.0011); unmarked coefficients did not survive correction in the text.

**Figure 3:**
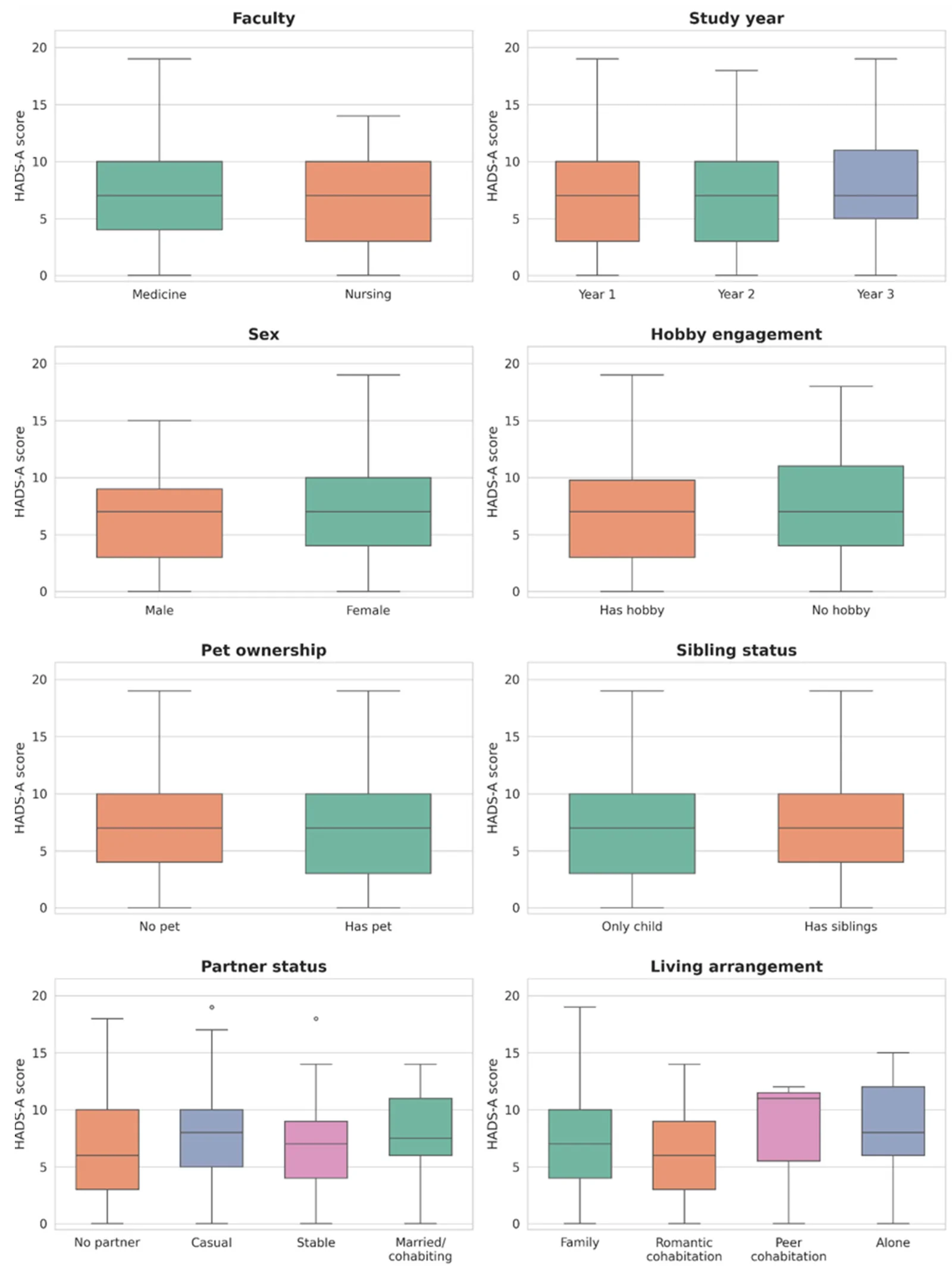
Boxplots of HADS-A anxiety scores by faculty, study year, sex, hobby engagement, pet ownership, sibling status, partner status, and living arrangement.

**Figure 4:**
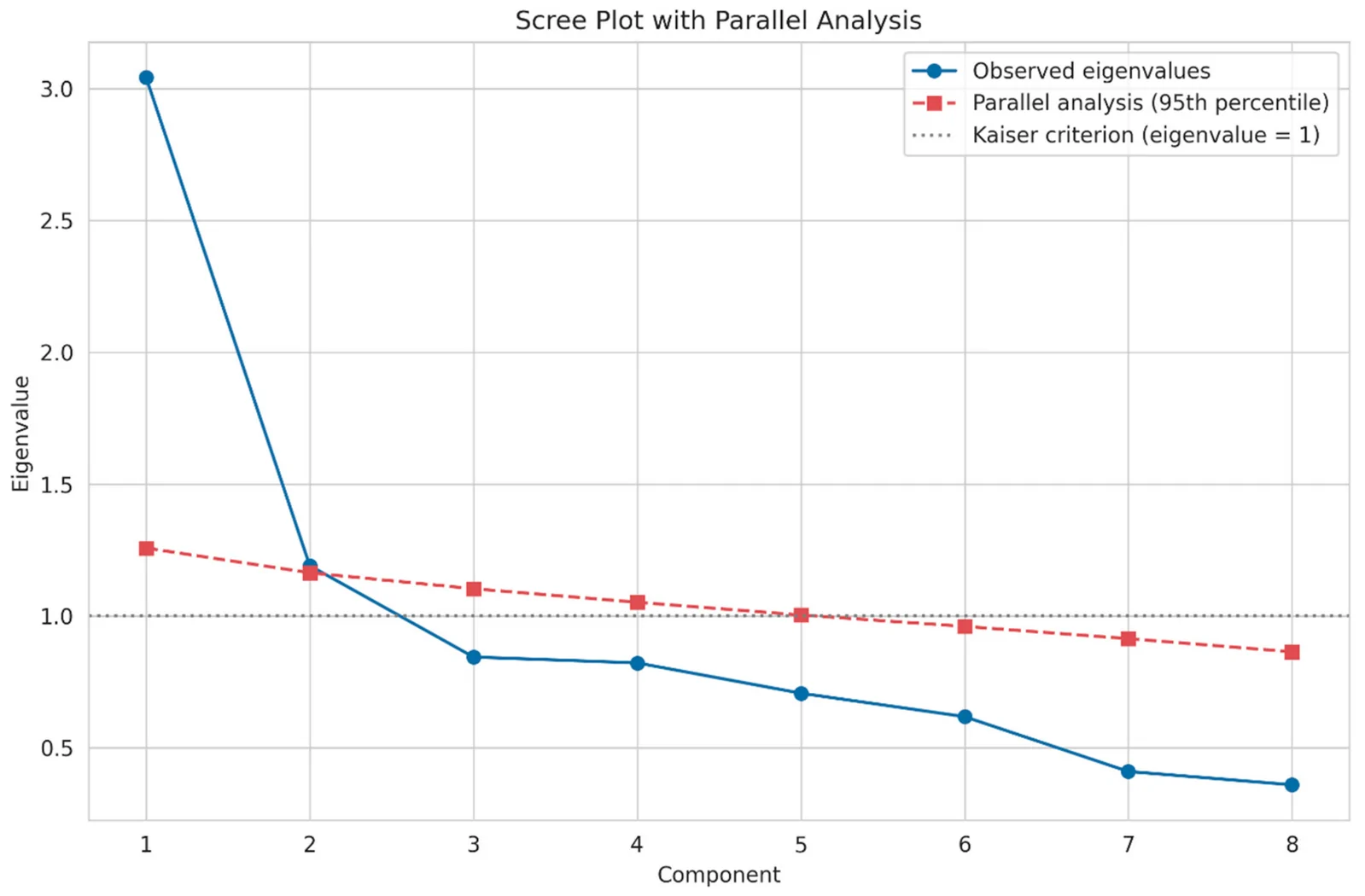
Scree plot with parallel analysis. Eigenvalues from observed data (solid line) cross the 95th-percentile parallel-analysis line (dashed) after the second component, supporting a two-component solution.

**Figure 5:**
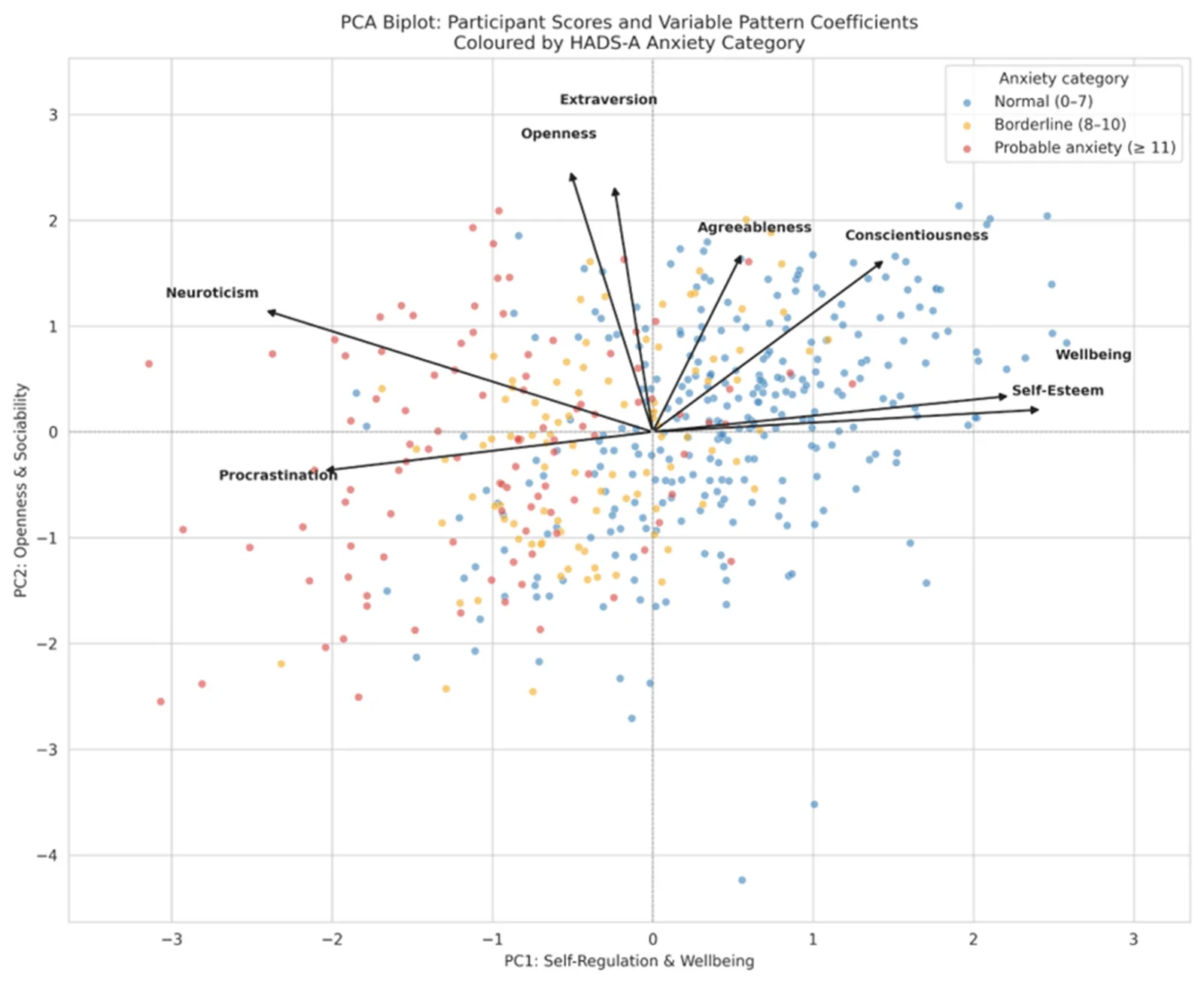
PCA biplot showing participant scores on PC1 (Self-Regulation & Wellbeing) and PC2 (Openness & Sociability), with variable pattern-coefficient arrows superimposed. Dots are coloured by HADS-A anxiety category.

**Figure 6:**
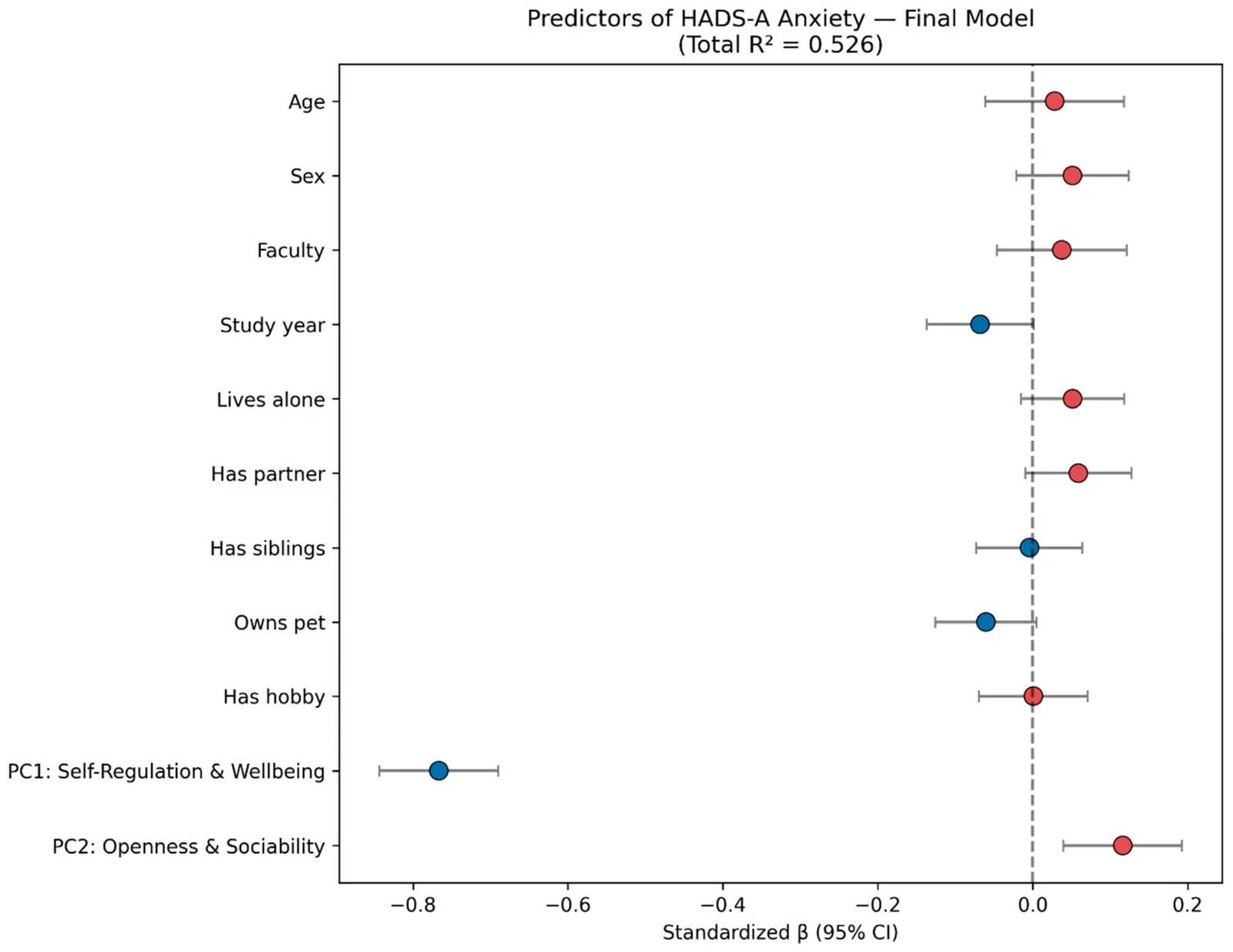
Standardised regression coefficients (β) with 95% confidence intervals for the final hierarchical regression model.

**Figure 7:**
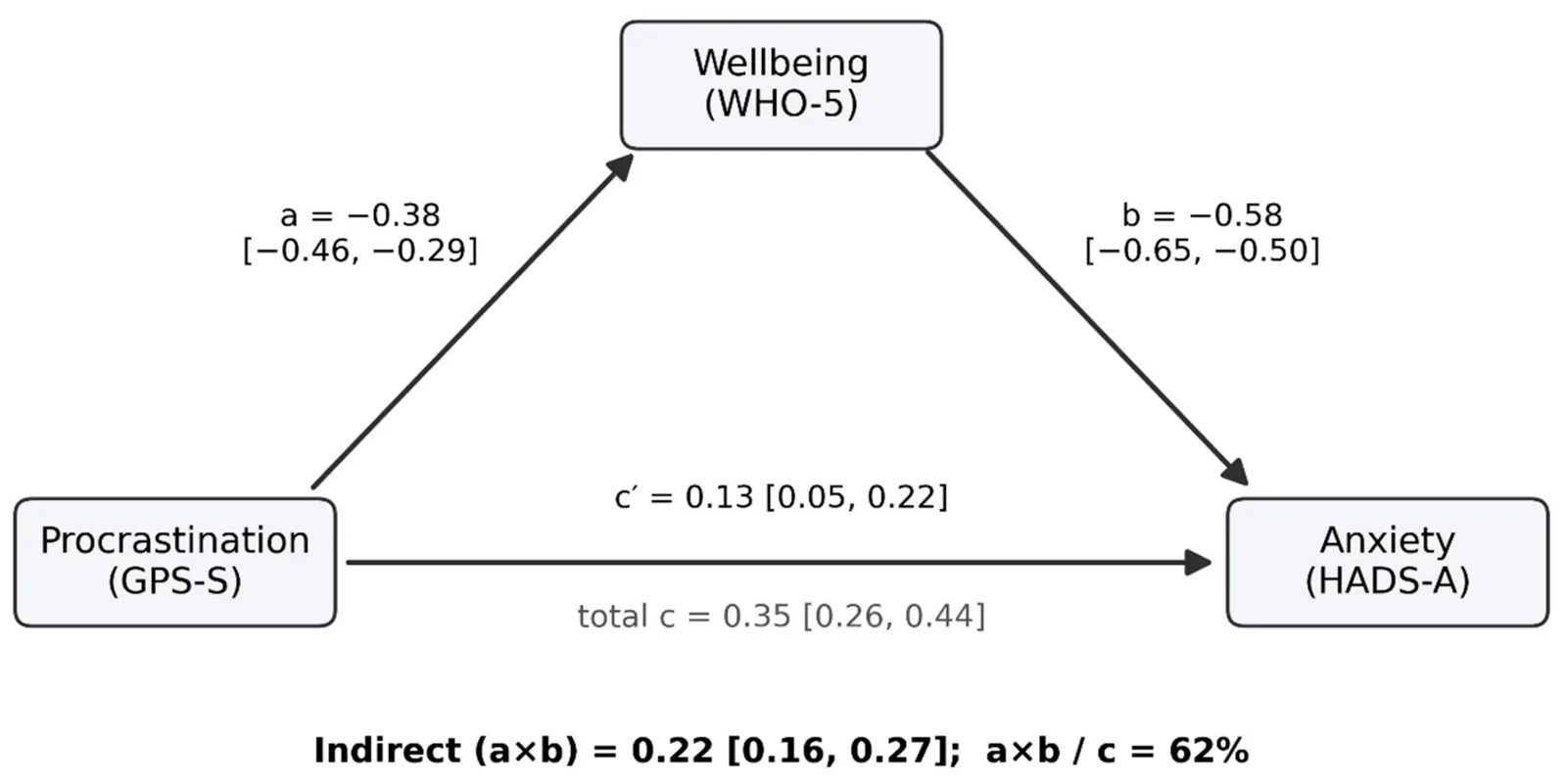
Exploratory mediation model; standardised path coefficients with 95% confidence intervals from bootstrap standard errors pooled across 20 imputed datasets. Arrows denote the tested statistical model, not temporal ordering.

**Figure 8:**
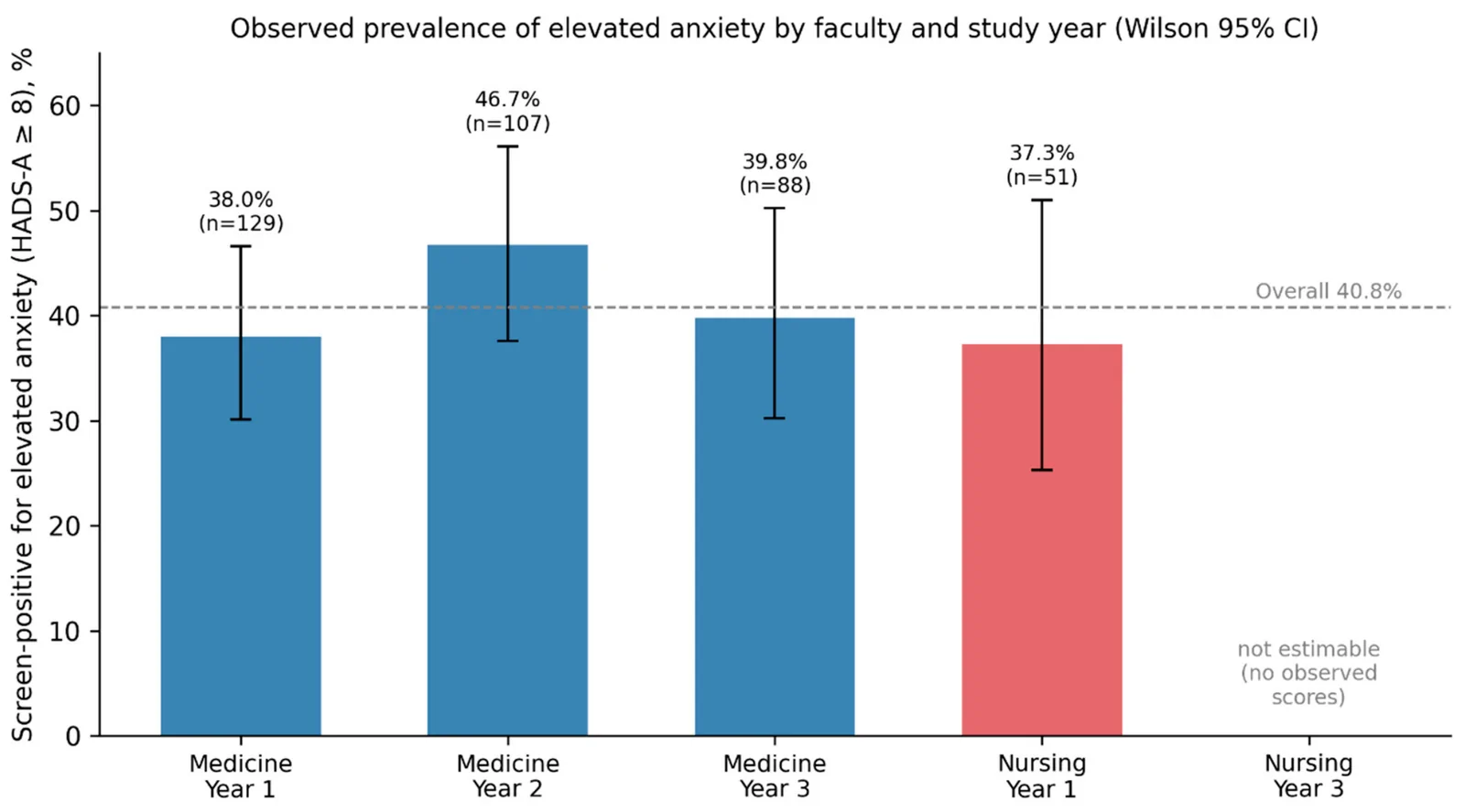
Prevalence of elevated anxiety (HADS-A ≥ 8) by faculty and study year.

**Figure 9:**
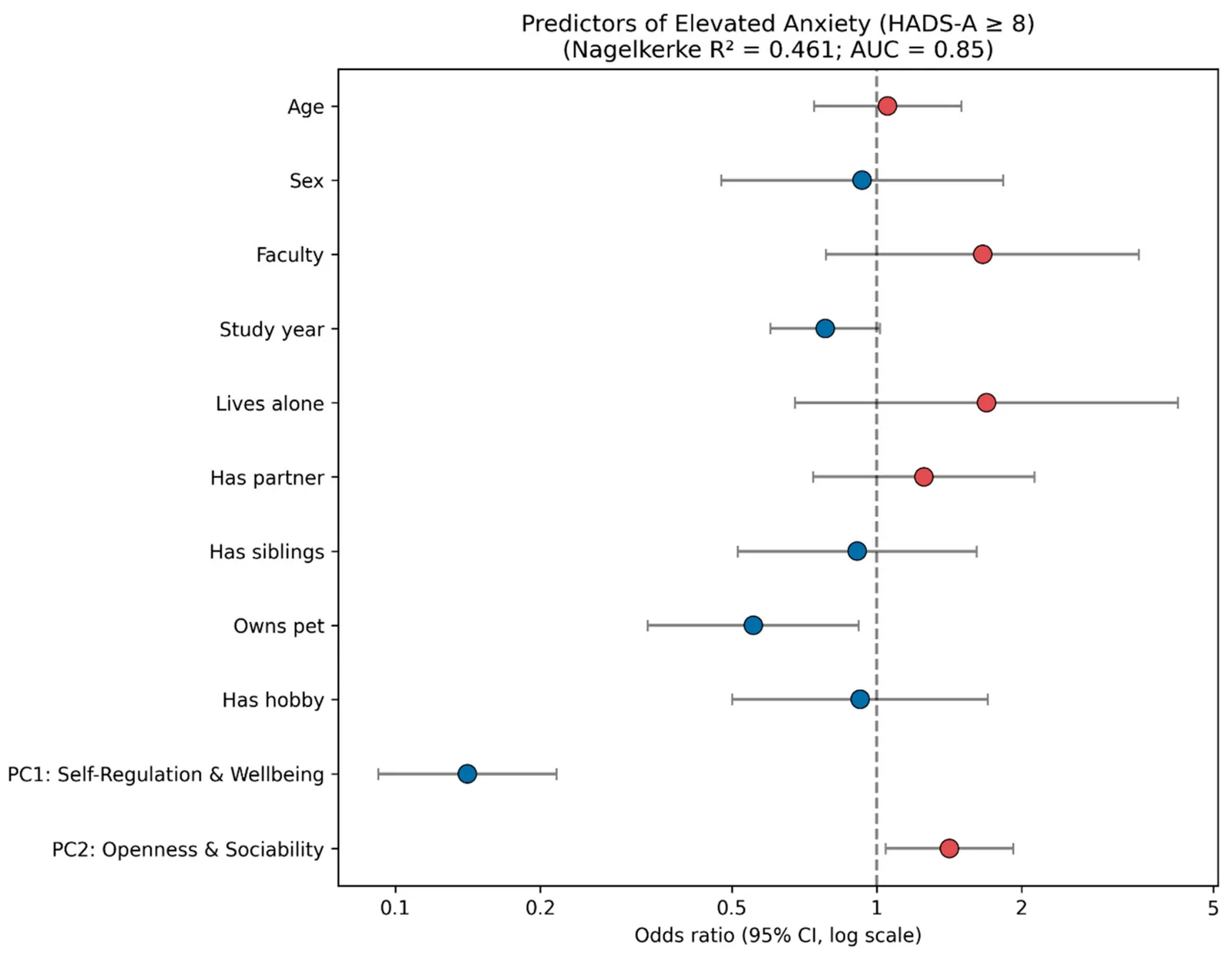
Odds ratios with 95% confidence intervals for the pooled logistic regression of elevated anxiety on the full predictor set.

**Table 1:** Sociodemographic, lifestyle, and HADS-A category characteristics (observed data).

Variable (n Observed)	Category	*n*	%
Faculty (n = 483)	Medicine	364	75.4
	Nursing	119	24.6
Study year (n = 483)	Year 1	209	43.3
	Year 2	113	23.4
	Year 3	161	33.3
Sex (n = 482)	Female	372	77.2
	Male	110	22.8
Living arrangement (n = 472)	With family	319	67.6
	Romantic cohabitation	110	23.3
	Peer cohabitation	3	0.6
	Alone	40	8.5
Sibling status (n = 474)	Has siblings	326	68.8
	Only child	148	31.2
Pet ownership (n = 473)	Has pet	281	59.4
	No pet	192	40.6
Romantic partner (n = 472)	No partner	305	64.6
	Casual relationship	50	10.6
	Stable relationship	45	9.5
	Married/cohabiting	72	15.3
Hobby engagement (n = 311)	Has hobby	211	67.8
	No hobby	100	32.2
HADS-A category (n = 375)	Normal (0–7)	222	59.2
	Borderline (8–10)	72	19.2
	≥11 (probable anxiety, screening range)	81	21.6

Denominators reflect the number of cases with that variable observed. Percentages within each variable are computed over the observed cases for that variable. Living arrangement was a multiple-response item; the 22 students who selected more than one option were assigned to a single category by the priority rule described in Section 2.5.

**Table 2:** Observed descriptive statistics for continuous measures.

Measure	*n*	M	SD	Range	Skewness	Kurtosis
Age (years)	477	25.12	9.61	18–54	1.44	0.68
Procrastination (GPS-S)	466	46.86	10.02	19–88	0.22	0.41
Openness	443	5.23	1.17	1.00–7.00	−0.55	0.30
Conscientiousness	443	5.30	1.04	2.00–7.00	−0.22	−0.54
Extraversion	443	4.73	1.16	1.00–7.00	−0.31	−0.06
Agreeableness	443	5.41	1.10	1.00–7.00	−0.84	0.83
Neuroticism	443	4.19	1.33	1.00–7.00	0.00	−0.47
Self-esteem (RSES)	429	20.82	5.73	0–30	−0.71	0.54
Wellbeing (WHO-5)	412	65.11	19.79	0–100	−0.57	−0.07
HADS-A anxiety	375	6.84	4.24	0–19	0.37	−0.48

All statistics are computed on observed (non-imputed) data. Values outside the theoretical range of each instrument (two procrastination scores > 90, two RSES scores > 30, one HADS-A score > 21) and five age values below 18 were recoded to missing prior to all analyses, including imputation. Statistics shown here are after these recodings.

**Table 3:** Pooled group comparisons of HADS-A anxiety scores across categorical variables (m = 20 imputed datasets).

Comparison	Test	Statistic	*p*	Effect Size	Mean Difference [95% CI]
Faculty (Medicine vs. Nursing)	Welch’s t	t(141.2) = +0.92	0.360	d = +0.10	0.42 [−0.49, 1.33]
Study year (1 vs. 2 vs. 3)	One-way ANOVA	F(2, 464.3) = 1.04	0.355	η^2^ = 0.008	-
Sex (Male vs. Female)	Welch’s t	t(196.4) = −1.78	0.077	d = −0.18	−0.76 [−1.59, 0.08]
Hobby engagement (yes vs. no)	Welch’s t	t(172.0) = −1.84	0.068	d = −0.20	−0.83 [−1.72, 0.06]
Pet ownership (yes vs. no)	Welch’s t	t(463.9) = +1.08	0.282	d = +0.10	0.42 [−0.34, 1.17]
Sibling status (only vs. has)	Welch’s t	t(242.1) = +0.36	0.717	d = +0.04	0.16 [−0.70, 1.02]
Partner status (4 levels)	One-way ANOVA	F(3, 235.0) = 0.86	0.465	η^2^ = 0.017	-
Living arrangement (4 levels)	One-way ANOVA	F(3, 138.4) = 1.17	0.324	η^2^ = 0.032	-

All comparisons are non-significant at Bonferroni-corrected α = 0.0063.

**Table 4:** Promax-rotated pattern matrix (loadings ≥ |0.40| in bold).

Variable	PC1: Self-Reg & Wellbeing	PC2: Openness & Sociability
Wellbeing (WHO-5)	**0.76**	0.07
Self-Esteem (RSES)	**0.70**	0.11
Conscientiousness	**0.46**	**0.51**
Procrastination	**−0.65**	−0.12
Neuroticism	**−0.76**	0.36
Openness	−0.16	**0.78**
Extraversion	−0.08	**0.73**
Agreeableness	0.18	**0.53**
Eigenvalue (unrotated)	3.04	1.19
% of total variance (unrotated)	38.0%	14.9%
Cumulative %	38%	52.9%

**Table 6:** Exploratory mediation model: standardised path coefficients with bootstrap standard errors pooled across 20 imputed datasets (Rubin’s rules).

Path	Estimate	SE	95% CI	*p*
a: Procrastination → Wellbeing	−0.38	0.04	[−0.46, −0.29]	<0.001
b: Wellbeing → Anxiety (adjusted for procrastination)	−0.58	0.04	[−0.65, −0.50]	<0.001
c: Total, Procrastination → Anxiety	0.35	0.05	[0.26, 0.44]	<0.001
c′: Direct, Procrastination → Anxiety	0.13	0.04	[0.05, 0.22]	0.003
a × b: Indirect	0.22	0.03	[0.16, 0.27]	<0.001
a × b/c	61.8%			

2000 bootstrap resamples per imputed dataset; arrows denote the tested statistical model, not temporal ordering.

**Table 7:** Prevalence of elevated anxiety (HADS-A ≥ 8) by faculty and study year: observed cases (Wilson 95% CI) and pooled estimates across 20 imputed datasets.

Group	N	n Observed	Observed % (95% CI)	Imputed % (95% CI)
All	483	375	40.8 (35.9–45.8)	43.0 (38.4–47.5)
Medicine Y1	140	129	38.0 (30.1–46.6)	40.0 (31.7–48.3)
Medicine Y2	113	107	46.7 (37.6–56.1)	46.7 (37.3–56.0)
Medicine Y3	111	88	39.8 (30.2–50.2)	44.2 (34.5–53.9)
Nursing Y1	69	51	37.3 (25.3–51.0)	38.3 (25.3–51.2)
Nursing Y3	50	0	not estimable	-

The Nursing programme did not include second-year students. No third-year Nursing student had an observed HADS-A score; the imputed estimate for this subgroup is not reported.

**Table 8:** Pooled logistic regression: odds ratios for elevated anxiety (HADS-A ≥ 8).

Predictor	OR	95% CI	*p*
Age	1.05	[0.74, 1.50]	0.772
Sex (Female)	0.93	[0.48, 1.83]	0.838
Faculty (Nursing)	1.66	[0.79, 3.51]	0.183
Study year	0.78	[0.60, 1.02]	0.065
Lives alone	1.69	[0.68, 4.23]	0.260
Has partner	1.25	[0.74, 2.13]	0.403
Has siblings	0.91	[0.52, 1.61]	0.747
Has pet	0.55 *	[0.34, 0.92]	0.022
Has hobby	0.92	[0.50, 1.70]	0.799
PC1: Self-Regulation & Wellbeing	0.14 ***	[0.09, 0.22]	<0.001
PC2: Openness & Sociability	1.42 *	[1.04, 1.92]	0.026

Standardised continuous predictors (age, year, PCA components); dummies otherwise. Nagelkerke R^2^ = 0.461 (mean across imputations); AUC = 0.85. * *p* < 0.05, *** *p* < 0.001.

## Data Availability

The de-identified participant-level dataset (total scores for all variables used in the analyses; names, dates of birth and any other identifiers removed) is deposited in the Zenodo repository under restricted access (https://doi.org/10.5281/zenodo.21723298). Access is granted by the corresponding author to researchers upon reasonable request, for verification or replication purposes, because the data were collected from students of a single institution and were not consented for unrestricted public release. The complete analysis code (Python scripts for data cleaning, scale scoring, multiple imputation with the settings described in Section 2.6.2, principal component analysis, all regression, mediation and sensitivity analyses, and the generation of every table and figure), together with a data dictionary of the variables in the deposited dataset, is available from the corresponding author on request.

## Supplementary Materials

The following supporting information can be downloaded at: https://www.mdpi.com/article/10.3390/healthcare14203455/s1. Table S1: Missing data by variable and programme–year group; Table S2: Participants with and without an observed HADS-A score; Table S3: Prevalence of elevated anxiety by faculty and study year, observed versus imputed; Table S4: Sensitivity analysis of the hierarchical regression model (full imputed sample, observed-outcome subsample, complete cases); Table S5: Sensitivity analysis of the logistic regression model (full imputed sample, observed-outcome subsample, complete cases); Table S6: Principal component analysis: pattern and structure coefficients, communalities and component correlations; Table S7: Hierarchical multiple regression of HADS-A scores, all blocks; Table S8: Final regression model with study year as a categorical predictor; Table S9: Regression diagnostics across imputed datasets; Table S10: Performance of the logistic regression model; Table S11: Covariate-adjusted exploratory mediation model; Table S12: Internal consistency of all scales in the present sample; Table S13: Participant flow; Table S14: Sensitivity analysis with the original psychological scales as separate predictors; Table S15: Final regression model with HC3 robust standard errors; Figure S1: Regression diagnostic plots for the final model; Supplementary Material S16: Romanian version of the General Procrastination Scale as administered.

## Abbreviations

The following abbreviations are used in this manuscript:

BFI-S	Big Five Inventory—Short Form
GPS-S	General Procrastination Scale, Student Version
HADS-A	Hospital Anxiety and Depression Scale, Anxiety Subscale
PHQ-9	Patient Health Questionnaire-9
SBI-U9	School Burnout Inventory—University version (9 items)
RSES	Rosenberg Self-Esteem Scale
WHO-5	WHO-5 Wellbeing Index
