# Supplementary material for: Psychological, Sociodemographic, and Lifestyle Correlates of Anxiety in Romanian Medical and Nursing Students: A Cross-Sectional Study
Source: Healthcare (Basel). 2026 Oct 15;14(20):3455. doi: 10.3390/healthcare14203455 (PMC13704339; doi:10.3390/healthcare14203455)
Supplement: Supplementary file 1 [file healthcare-14-03455-s001.zip › Supplementary_Material_S16_GPS_Romanian_version.pdf]

Supplementary Material S16. General Procrastination Scale – Romanian version as administered

Manuscript: Psychological, Sociodemographic, and Lifestyle Correlates of Anxiety in Romanian Medical and Nursing Students: A Cross-Sectional Study (Healthcare, manuscript 4553953).

Source instrument: Lay, C.H. At Last, My Research Article on Procrastination. J. Res. Pers. 1986, 20, 474–495 (20 items, reference 26 of the manuscript). The Romanian wording was translated from English by two members of the research team (A.P.-C. and C.P.-C.), without back-translation, cognitive debriefing, pilot testing or formal validation (Section 2.4.7). Owing to an error in building the online questionnaire in PsyToolkit, items 15 and 17 of the original scale were omitted; the omission was noticed only after data collection had ended. The 18 administered items are reproduced below exactly as presented to participants, in the order administered, together with the original English item and the original item number. Items marked R were reverse-keyed according to the original key. Responses were given on a 5-point scale (1 = extremely uncharacteristic of me, 5 = extremely characteristic of me) and summed to a total score (theoretical range 18–90), with higher scores indicating greater procrastination.

No. administered	Original item no. (Lay, 1986)	Romanian item as administered	Original English item	Key
1	1	De multe ori mă trezesc îndeplinind sarcini pe care intenționam să le fac cu câteva zile înainte.	I often find myself performing tasks that I had intended to do days ago.	
2	2	Cand am ceva de facut, nu ma apuc de treaba decat in ultimul moment.	I do not do assignments until just before they are to be handed in.	
3	3	Când termin cu o carte de bibliotecă, o returnez imediat, indiferent de data scadenței.	When I am finished with a library book, I return it right away regardless of the date it is due.	R
4	4	Când este timpul să mă trezesc dimineața, cel mai adesea mă ridic imediat din pat.	When it is time to get up in the morning, I most often get right out of bed.	R
5	5	O scrisoare (email) poate astepta câteva zile după ce o scriu înainte de a o trimite prin poștă.	A letter may sit for days after I write it before mailing it.	
6	6	În general, răspund apelurile telefonice prompt.	I generally return phone calls promptly.	R
7	7	Chiar și lucrurile foarte usoare, pe care le pot face rapid, deseori le aman zile intregi	Even with jobs that require little else except sitting down and doing them, I find they seldom get done for days.	
8	8	De obicei iau o decizie cât mai repede posibil.	I usually make decisions as soon as possible.	R
9	9	În general, întârzii înainte de a ma apuca de munca pe care trebuie să o fac.	I generally delay before starting on work I have to do.	
10	10	De obicei trebuie să mă grăbesc să termin o sarcină la timp.	I usually have to rush to complete a task on time.	
11	11	Când mă pregătesc să ies undeva, rareori se intampla că trebuie să fac ceva în ultimul moment.	When preparing to go out, I am seldom caught having to do something at the last minute.	R
12	12	Pregătindu-mă pentru un anumit termen limită, deseori pierd timpul făcând alte lucruri.	In preparing for some deadline, I often waste time by doing other things.	

13	13	Prefer să plec devreme la o întâlnire.	I prefer to leave early for an appointment.	R
14	14	Cand mi se da ceva de facut de obicei ma apuc de treaba imediat.	I usually start an assignment shortly after it is assigned.	R
15	16	De obicei cadourile de craciun sau de ziua cuiva le cumpar în ultimul moment.	I always seem to end up shopping for birthday or Christmas gifts at the last minute.	
16	18	De obicei, realizez toate lucrurile pe care plănuiesc să le fac într-o zi.	I usually accomplish all the things I plan to do in a day.	R
17	19	Spun continuu „O voi face mâine”.	I am continually saying "I'll do it tomorrow."	
18	20	De obicei mă ocup rezolv toate sarcinile pe care trebuie sa le fac inainte de a ma aseza si a ma relaxa seara	I usually take care of all the tasks I have to do before I settle down and relax for the evening.	R

Items of the original scale not administered:

Item 15 (R): I often have a task finished sooner than necessary.

Item 17: I usually buy even an essential item at the last minute.

R = reverse-keyed item (scored 6 − response). Nine of the 18 administered items are reverse-keyed (original items 3, 4, 6, 8, 11, 13, 14, 18 and 20); of the two omitted items, item 15 is reverse-keyed and item 17 is not.
